# The interrelationship between multiple long-term conditions (MLTC) and delirium: a scoping review

**DOI:** 10.1093/ageing/afae120

**Published:** 2024-07-04

**Authors:** Sarah Joanna Richardson, Alexandria Danielle Cropp, Samantha Wilhelmina Ellis, Jake Gibbon, Avan Aihie Sayer, Miles David Witham

**Affiliations:** AGE Research Group, Translational and Clinical Research Institute, Faculty of Medical Sciences, Newcastle University, Newcastle upon Tyne, Tyne and Wear, UK; NIHR Newcastle Biomedical Research Centre, Newcastle upon Tyne Hospitals NHS Foundation Trust, Cumbria Northumberland Tyne and Wear NHS Foundation Trust and Faculty of Medical Sciences Newcastle University, Newcastle upon Tyne, Tyne and Wear, UK; Northumbria Healthcare NHS Foundation Trust, Newcastle upon Tyne, Tyne and Wear, UK; Northumbria Healthcare NHS Foundation Trust, Newcastle upon Tyne, Tyne and Wear, UK; South Tyneside and Sunderland NHS Foundation Trust, South Shields, Tyne and Wear, UK; AGE Research Group, Translational and Clinical Research Institute, Faculty of Medical Sciences, Newcastle University, Newcastle upon Tyne, Tyne and Wear, UK; NIHR Newcastle Biomedical Research Centre, Newcastle upon Tyne Hospitals NHS Foundation Trust, Cumbria Northumberland Tyne and Wear NHS Foundation Trust and Faculty of Medical Sciences Newcastle University, Newcastle upon Tyne, Tyne and Wear, UK; AGE Research Group, Translational and Clinical Research Institute, Faculty of Medical Sciences, Newcastle University, Newcastle upon Tyne, Tyne and Wear, UK; NIHR Newcastle Biomedical Research Centre, Newcastle upon Tyne Hospitals NHS Foundation Trust, Cumbria Northumberland Tyne and Wear NHS Foundation Trust and Faculty of Medical Sciences Newcastle University, Newcastle upon Tyne, Tyne and Wear, UK

**Keywords:** delirium, multiple long-term conditions, scoping review, epidemiology, older people

## Abstract

**Introduction:**

Delirium and multiple long-term conditions (MLTC) share numerous risk factors and have been shown individually to be associated with adverse outcomes following hospitalisation. However, the extent to which these common ageing syndromes have been studied together is unknown. This scoping review aims to summarise our knowledge to date on the interrelationship between MLTC and delirium.

**Methods:**

Searches including terms for delirium and MLTC in adult human participants were performed in PubMed, EMBASE, Medline, Psycinfo and CINAHL. Descriptive analysis was used to summarise findings, structured according to Synthesis Without Meta-analysis reporting guidelines.

**Results:**

After removing duplicates, 5256 abstracts were screened for eligibility, with 313 full-texts sought along with 17 additional full-texts from references in review articles. In total, 140 met inclusion criteria and were included in the final review. Much of the literature explored MLTC as a risk factor for delirium (*n* = 125). Fewer studies explored the impact of MLTC on delirium presentation (*n* = 5), duration (*n* = 3) or outcomes (*n* = 6) and no studies explored how MLTC impacts the treatment of delirium or whether having delirium increases risk of developing MLTC. The most frequently used measures of MLTC and delirium were the Charlson Comorbidity Index (*n* = 98/140) and Confusion Assessment Method (*n* = 81/140), respectively.

**Conclusion:**

Existing literature largely evaluates MLTC as a risk factor for delirium. Major knowledge gaps identified include the impact of MLTC on delirium treatment and the effect of delirium on MLTC trajectories. Current research in this field is limited by significant heterogeneity in defining both MLTC and delirium.

## Key points

- Existing literature largely evaluates multiple long-term conditions (MLTC) as a risk factor for the prevalence of delirium rather than the presentation, duration or outcomes of delirium.
- No studies explore the impact of MLTC on the treatment of delirium.
- No studies identify whether delirium increases the risk of developing MLTC.
- Methodological heterogeneity in defining MLTC and delirium is limiting the field.

## Introduction

The term multiple long-term conditions (MLTC) refers to the existence of two or more chronic conditions in a single individual [1]. MLTC replaces the term ‘multimorbidity’, in line with a recent report which found that ‘multimorbidity’ was considered a negative term by people living with MLTC, who also did not think the term was an accurate or useful description of their conditions [2]. The presence of MLTC is strongly associated with age and deprivation, and people living with MLTC experience worse quality of life and reduced life expectancy [3, 4]. Despite being identified as a research priority globally, existing evidence regarding the epidemiology of MLTC has been limited by the absence, until recently, of an agreed definition for MLTC [1, 5].

MLTC has been shown to increase the risk of hospitalisation and, once hospitalised, people living with MLTC often stay longer and experience greater treatment burden [3]. Much of the existing MLTC literature has focused on primary care populations and evidence regarding the experience and outcomes of people living with MLTC admitted to hospital is limited. Gaining insights into the effectiveness of health services and healthcare utilisation for people living with MLTC, and how to design services to better serve people living with MLTC, have been identified as particular research priorities [1, 6, 7].

Delirium is a syndrome characterised by acute confusion, inattention and altered arousal [8]. It affects at least 20% of hospital inpatients, with higher occurrence rates in older people and those with cognitive impairment [9]. As well as being highly distressing, delirium is associated with many of the same adverse outcomes seen in people living with MLTC, including increased length of stay, additional healthcare costs and increased mortality [10–12].

As both delirium and MLTC are common and associated with adverse consequences, it is important to understand how these clinical challenges are interrelated. A key first step is to understand the range of existing literature regarding the interrelationship between MLTC and delirium. Therefore, a scoping review is appropriate to capture the breadth and extent of existing research, and to identify the gaps in the research landscape. The review aims to describe existing literature on the topic of MLTC and delirium, while drawing attention to strengths and limitations of existing studies and highlighting gaps in our knowledge. The specific aims for this review were to describe (a) studies that have examined the impact of MLTC on delirium prevalence, presentation and treatment in hospitalised patients, (b) studies that have examined the contribution of both delirium and MLTC to outcomes of hospitalised patients and (c) how delirium and MLTC have been defined in studies examining both.

## Methods

A pre-specified protocol was developed for this scoping review; the review has been registered and the protocol published on the Open Science Framework platform (https://osf.io/9tqyh).

### Eligibility criteria

Studies published from inception of each database until the date of searches (16 November 2023) were eligible for inclusion. Studies were limited to human studies published in English. All study designs were included except case reports, narrative reviews, study protocols and conference abstracts along with other non-journal articles (e.g. book chapters). We limited studies to those including adults (aged 18 and over), excluding studies of paediatric delirium. Studies that included a measure of MLTC only as an analytic adjustment variable (rather than as a focus of analysis) were excluded.

All the following three criteria had to be met for a report to be included when assessing eligibility of full texts.

1. Included hospitalised patients with delirium, diagnosed using a recognised and validated method based upon criteria for delirium within iterations of the Diagnostic and Statistical Manual (DSM) or International Classification of Diseases (ICD).
2. Recorded MLTC/multiple comorbidities/multimorbidity using the definition of two or more long-term conditions OR a recognised measure of comorbidity (e.g. Charlson comorbidity index (CCI), Cumulative Illness Rating Scale (CIRS), disease counts).
3. Examined how MLTC impacted on prevalence, presentation, duration or treatment of delirium OR examined the contribution of both delirium and MLTC to outcomes, including the impact of delirium on the development of MLTC.

### Information sources

Searches took place on 16 November 2023 of PubMed, EMBASE, Medline, Psycinfo and CINAHL. Searches included terms for delirium AND MLTC, and were designed to be broad to capture the scope of existing literature. The reference list of retrieved review articles were hand-searched to find additional papers. The search strategy was based upon the strategies used by the National Institute for Health and Care Excellence (NICE) delirium guidance and a recent multimorbidity review [13, 14]. The full search strategy for all databases is shown in Supplementary Material.

### Selection of sources of evidence

All search results were combined and exported into Zotero Version 6 (2022) to remove duplicates [15]. References were then exported to Rayyan (2022) for screening [16]. A minimum of two reviewers [S.R., A.C., S.E., J.G.] independently screened each title and abstract for eligibility and relevance and rated each title as ‘Include’ or ‘Exclude’. All conflicts were reviewed following unblinding and were resolved by consensus. If any reviewer felt a reference should be included or was uncertain, it went into the next stage of adjudication. Full texts of eligible abstracts were sought to confirm eligibility by two independent reviewers [S.R., A.C., J.G.]. If, at any stage, consensus could not be met, a third independent reviewer adjudicated [M.W.].

### Data charting process

Following confirmation of eligibility, data were extracted by a single researcher and checked by a second researcher [S.R., A.C.]. Data were charted using a proforma designed specifically for this scoping review (Supplementary Material). This contained fields including year of publication, setting, number and age of participants, methodology used for delirium and MLTC diagnosis, main objectives of the study, key findings and funding information.

### Assessment of bias in individual sources of evidence

Due to the wide variety of study types included, formal assessment of bias was not undertaken.

### Synthesis of results

Descriptive analysis was used to summarise the findings, structured according to Synthesis Without Meta-analysis (SWiM) reporting guidelines [17]. Studies were grouped according to their main findings, structured according to the pre-specified aims. Systematic reviews were included in order to capture and describe the scope of the literature available to date on this topic but were excluded when synthesising results for individual studies to avoid duplication.

## Results

**Figure 1:**
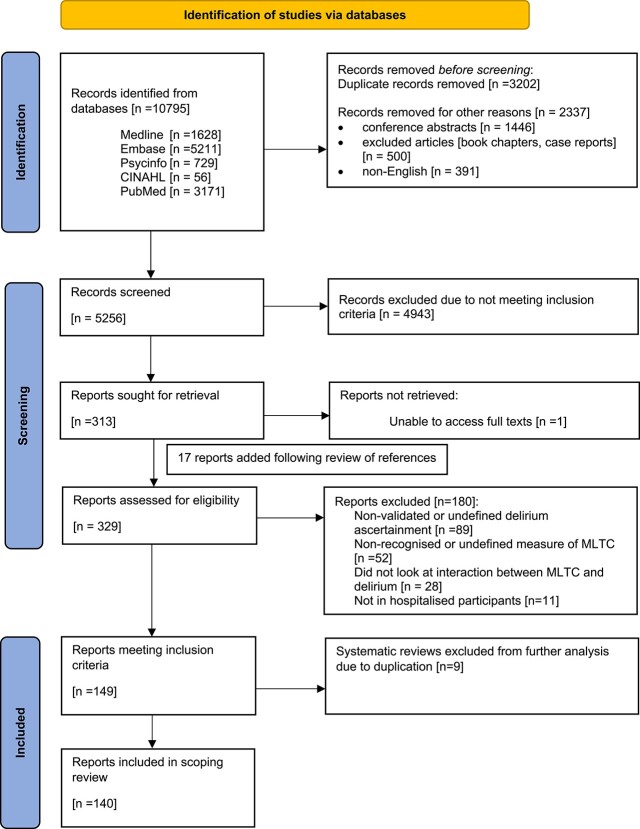
Search results.

Following combining and de-duplication of search results, 5256 abstracts remained and were screened for eligibility. A total of 313 full texts were sought along with 17 additional full texts added following hand-searching of references in review articles. Of these, 329 were available and assessed for eligibility, with 140 articles meeting the eligibility criteria and included in the final review (Figure 1) (Supplementary Table 1).

**Figure 2:**
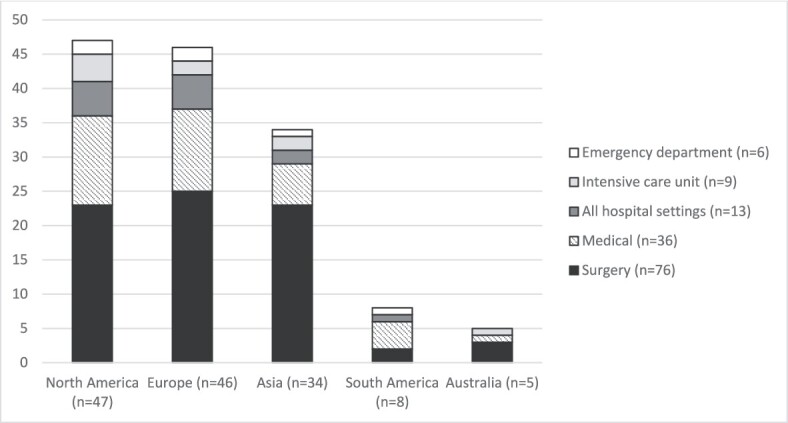
Geographical location and setting of included articles.

The most frequently included geographical settings were North America (*n* = 47, 34%), Europe (*n* = 46, 33%) and Asia (*n* = 34, 24%). In total, 26 were conducted in countries defined as low or middle income [18]. The most frequently studied setting was surgery (*n* = 76, 54%), followed by medicine (*n* = 36, 26%) (Figure 2).

Nine systematic reviews met the eligibility criteria but were not included in further analysis to avoid duplication. These systematic reviews all focused on investigating risk factors for delirium, with one focusing specifically on risks factors for persistent delirium [19]. Further details of these are included within Supplementary Table 2.

### Impact of MLTC on delirium prevalence

In total, 125 reports explored the role of MLTC as a risk factor for delirium. Of these, 40 found no significant relationship between MLTC and delirium, while 85 (68.0%) concluded that MLTC was a risk factor for delirium; 77 in univariate analysis and 32 studies found that this relationship remained significant after adjusting for other variables. The methods of recording MLTC and delirium were similar across these studies with the majority using CCI to record MLTC and CAM (Confusion Assessment Method) to diagnose delirium (Table 1) (Supplementary Material Figure 1a and b). A higher proportion of studies showing significant findings were in the top quartile of study size (sssSupplementary Material Figure 1d).

**Table 1:** Summary of 125 included reports presenting data on MLTC as a risk factor for delirium. For further details regarding the studies mentioned within this table, including a full list of the variables including in multivariate analysis, see Supplementary Table 1

**Author, year**	**Sample size** **(% with delirium)**	**Setting**	**Measure of MLTC**	**Measure of delirium**	**MLTC as a risk factor for delirium**			
					**Univariate analysis performed**	**Significant in univariate analysis**	**Multivariate analysis performed**	**Significant in multivariate analysis**
CAM used to measure delirium								
Alamri, 2018 [20]	147 (21.8%)	Older medical patients ≥60 years old, Saudi Arabia	Number of comorbidities (0–2; 3–5)	CAM (on admission)	x	x	x	x
Freter, 2016 [21]	283 (57.6% pre-surgery; 41.7% post-surgery)	Hip fracture patients >65 years, Canada	Number of comorbidities	CAM (pre-operatively then post-operative days 1, 3 and 5)	x	x		
Goldenburg, 2006 [22]	77 (48.1%)	Hip fracture patients >65 years old, USA	Number of comorbidities (expressed as a morbidity index)	CAM (daily)	x	x	x	
Lee, 2011 [23]	425 (35%)	Hip fracture surgery patients >65 years old, USA	Number of comorbidities	CAM (daily)	x	x	x	x
Lochanie, 2018 [24]	30 (66.7%)	Mechanically ventilated patients in surgical ICU, Sri Lanka	Number of comorbidities (≥2)	CAM-ICU (daily)	x			
Mangnall, 2011 [25]	118 (35%)	≥50 years, admitted for elective major colorectal surgery, Australia	Number of comorbidities	CAM (daily for first 3 days post-operatively)	x			
Narayanan, 2022 [26]	50 (22%)	Post-operative cancer patients >60 years old, India	Number of comorbidities (≥3)	Short-CAM (twice daily for 3 days)	x	x		
Ng, 2019 [27]	280 (12.9%)	Consecutive patients with acute ischaemic stroke aged ≥18 years, Australia	Number of comorbidities (≥2 from pre-defined list of seven conditions)	CAM (within 7 days of stroke)	x			
Saljuqi, 2020 [28]	163 (26%)	≥65 years undergoing emergency general surgery, USA	Number of comorbidities (≥3)	CAM	x	x	x	x
Tognoni, 2011 [29]	90 (8.8%)	Consecutive patients undergoing urological surgery, Italy	Number of diseases (≥2)	CAM (daily for 7 days post-operatively)	x			
Wang, 2017 [30]	265 (18.5%)	≥65 years undergoing elective total knee arthroplasty, Republic of Korea	≥2 groups according to CCI	CAM (triggered by concerns about orientation)	x			
Witlox, 2011 [31]	76 (39.5%)	Hip fracture patients ≥75 years old, Netherlands	Number of comorbidities	CAM (daily until postoperative day 5)	x			
Xue, 2016 [32]	358 (7.8%)	≥65 years and undergoing TURP, China	Number of co-existent medical conditions ≥2	CAM (daily for 7 days post-operatively or if signs of confusion)	x	x	x	
Zhao, 2023 [33]	199 (28.10%)	≥90 years old with hip fracture, China	Number of comorbidities (≥4)	CAM (daily)	x	x	x	x
Afonso, 2010 [34]	112 (34%)	Cardiac surgery ICU, USA	CCI	CAM (every 12 hours)	x	x		
Alvarez, 2023 [35]	143 (9.10%)	≥75 years undergoing elective major surgery, Chile	CCI	CAM (twice a day for 5 days or until discharge)	x			
Arias, 2022 [36]	547 (23%)	Major, non-cardiac surgery patients ≥70 years old, USA	CCI	CAM (daily)			x	x
Béland, 2021 [37]	612 (11%)	Older emergency department patients ≥65 years old, Canada	Age adjusted CCI	CAM (twice daily)	x			
Billig, 2022 [38]	732 (13.5%)	Emergency department patients ≥60 years old, Brazil	CCI (divided into categories of survival)	CAM (on admission)	x	x	x	
Carrasco, 2014 [39]	374 (6.68%)	General medical inpatients ≥65 years old, Chile	CCI	CAM (every 48 hours)	x			
Cunningham, 2019 [40]	282 (14%)	Elective hip and knee patients ≥65 years old, Northern Ireland	CCI	CAM (daily for 3 days)	x	x	x	x
Dworkin, 2016 [41]	76 (13.2%)	Elective surgical patients ≥65 years old, USA	Charlson-Deyo	CAM (in hospital and FAM-CAM before)	x	x		
Eide, 2015 [42]	143 (53.1%)	Octogenarians undergoing elective TAVI or SAVR, Norway	CCI	CAM (daily for 5 days post operatively)	x			
Eschweiler, 2021 [43]	880 (23.6%)	Surgical patients >70 years old, Germany	CCI Number of diseases (≤4, 5–8, ≥9)	CAM (daily for 7 days)	x	x	x	x [number of diseases only]
Esmaeeli, 2022 [44]	556 (14%)	≥65 years old, orthopaedic trauma patients, USA	CCI	CAM (daily)	x	x	x	
Fick, 2000 [45]	20 (60%)	Inpatients >65 years, USA	CCI	CAM (daily)	x	x		
Flaherty, 2010 [46]	148 (29.7%)	Acute older persons ward ≥65 years old, USA	CCI	Modified CAM (daily for 6 days)	x			
Fortes-Filho, 2016 [47]	147 (41.5%)	Hip fracture patients, ≥60 years, Brazil	CCI (0; 1; 2; ≥3)	CAM (daily)	x			
Franz, 2023 [48]	5042 (37.10%)	All adult (≥18 years old) patients admitted to the medical ICU, USA	CCI	CAM-ICU (twice daily)			x	
Galanakis, 2001 [49]	105 (23.8%)	Elective and emergency hip surgery patients ≥60 years old, Germany	CCI	CAM (daily)	x	x	x	
Giuseffi, 2017 [50]	226 (20%)	TAVR or SAVR patients, USA	CCI	CAM-ICU (twice daily)			x	
Guenther, 2013 [51]	215 (31%)	Consecutive patients ≥50 years scheduled for cardiac surgery, Germany	CCI	CAM-ICU (daily for first 7 days post-operatively)	x	x	x	x
Guo, 2019 [52]	244 (24.6%)	Hip fracture surgery patients aged 65 to 80 years, China	CCI	CAM (twice daily for first 3 days post-operatively)	x			
Hamann, 2005 [53]	100 (7%)	≥60 year olds undergoing urologic surgery, Germany	CCI (>3)	CAM (daily)	x			
Hu, 2022 [54]	531 (23.5%)	Surgical patients, China	CCI	CAM-ICU or CAM (twice a day for 3 days post-operatively)			x	x
Janssen, 2019 [55]	627 (10.2%)	≥70 years, elective surgery for colorectal cancer or abdominal aortic aneurysm, Netherlands	CCI (≥7)	CAM or DSM5 (regularly)	x	x	x	
Juliebø, 2009 [56]	237 (21.1% pre-operative 36.4% post-operative)	≥65 years old, two orthopaedic surgery departments, Norway	CCI (>1)	CAM (daily for up to 5 days post-operatively or discharge)	x			
Kang, 2021 [57]	683 (18.3%)	Vascular surgery for peripheral vascular disease, Republic of Korea	CCI	CAM-ICU (daily)	x	x	x	x
Kennedy, 2014 [58]	676 (9%)	Emergency department patients >65 years, USA	CCI	CAM (once)	x	x		
Khan, 2022 [59]	408 (16.7%)	Acute medical unit patients ≥65 years, USA	CCI (continuous and >2)	3D CAM (daily)	x	x		
Kim, 2023 [60]	257 (20.2%)	>70 year olds undergoing spinal surgery, Republic of Korea	CCI	CAM (four times daily)	x	x	x	
Korc-Grodzicki, 2015 [61]	416 (19%)	Surgical patients with solid tumours aged ≥75 years, USA	CCI (≥3)	CAM (daily)	x	x	x	x
Lai, 2022 [62]	345 (5.5%)	≥65 years, elective surgery for gastrointestinal cancer, Taiwan	CCI (minus age and cancer) (≥3)	CAM (daily)	x	x	x	x
Large, 2013 [63]	49 (29%)	Radical cystectomy patients ≥65 years, USA	Age adjusted CCI	CAM (post-operative days 1,2,3,5,7)	x			
Lee, 2023 [64]	1353 (5.8%)	Consecutive patients undergoing hip bipolar hemiarthroplasty for displaced femoral neck fractures, Taiwan	CCI (≥6)	CAM	x	x	x	x
Leung, 2015 [65]	50 (14%)	≥40 years scheduled for major non-cardiac surgery, USA	CCI	CAM (daily for 3 days post-operatively)	x			
Li, 2015 [66]	38 (18.4%)	Non-delirious consecutive patients scheduled for an isolated CABG, Taiwan	CCI	CAM (daily for 1 week post-operatively)	x	x		
Liu, 2022 [67]	309 (16.8%)	Hip fracture patients >65 years, China	Age adjusted CCI	CAM (twice daily for 2 days)	x	x	x	x
Liu, 2022 [68]	184 (19.6%)	Thoracic and abdominal surgery patients ≥60 years, China	Age adjusted CCI	CAM (twice daily for 3 days)	x	x	x	x
Marcantonio, 2000 [69]	126 (41.3%)	Hip fracture patients >65 years, USA	CCI (≥4)	CAM (daily)	x	x		
McAlpine, 2008 [70]	103 (17.5%)	Gynaecological cancer patients ≥60 years, Canada	CCI	CAM (post-operative day 1)	x	x		
McCusker, 2004 [71]	318 (69.8%)	Medical patients ≥65 years, Canada	CCI	CAM (once)	x	x		
Moreno-Gaviño, 2012 [72]	1632 (11%)	Outpatients and hospital at home patients, Spain	CCI	CAM (during most recent hospital admission)	x	x		
Mossello, 2020 [73]	497 (18%)	Cardiac ICU patients >65 years, Italy	CCI	CAM-ICU (daily)	x	x	x	x
Naksuk, 2017 [74]	11 079 (8.3%)	Patients admitted to coronary care unit, USA	CCI	CAM-ICU (twice daily)	x	x		
Pioli, 2019 [75]	939 (31.1%)	Hip fracture patients ≥75 years, Italy	CCI	CAM (daily)	x	x	x	
Radinovic, 2015 [76]	270 (53%)	Hip fracture patients ≥60 years, Serbia	CCI (without age) (>1)	CAM (multiple times, at least daily)	x	x	x	
Radinovic, 2019 [77]	277 (53%)	>60 years with hip fracture, Serbia	CCI	CAM (daily for 7 days post-operatively)	x	x		
Ranhoff, 2006 [78]	401 (29.2%)	Medical unit patients ≥60 years, Italy	CCI	CAM (daily)	x	x		
Ritchie, 2014 [79]	710 (12.3%)	Medical Admissions Unit patients >70 years, UK	CCI	CAM (once within 72 hours of admission)	x			
Robinson, 2009 [80]	144 (44%)	>50 year olds scheduled for an operation requiring a post-operative ICU admission, USA	CCI	CAM-ICU (daily)	x	x	x	x
Romanauski, 2018 [81]	6338 (9.6%)	General anaesthetic patients admitted to ICU, USA	CCI (without age)	CAM-ICU (every 12 hours)	x	x	x	x
Singler, 2014 [82]	133 (14.3%)	Emergency Department patients ≥75 years old, Germany	CCI (≥3)	CAM – shortened form (once)	x			
Smith, 2015 [83]	63 (37%)	Lung transplant patients, USA	CCI	CAM (daily)	x			
Sugi, 2023 [84]	158 (33.5%)	Patients ≥75 years old undergoing elective surgery for gastro-enterological cancer, Japan	CCI (≥8)	CAM (three times daily)	x	x	x	
Tan, 2008 [85]	53 (23%)	Elective cardiac surgery patients, USA	CCI	CAM (daily for first 7 days post-op)	x	x		
Tkacheva, 2017 [86]	181 (7.2%)	Acute hospital patients ≥65 years old, Russia	CCI	CAM-ICU (once)	x	x		
Ansaloni, 2010 [87]	357 (13.2%)	General elective and emergency surgery patients >65 years old, Italy	CIRS (≥8)	CAM (on post-operative days 1, 2, 3, 6)	x	x	x	x
Ferré, 2022 [88]	67 (54%)	≥65 year olds undergoing urgent surgery for hip fracture, France	CIRS-G	CAM (three times daily until post-operative day 7 or discharge)	x			
Kimura, 2023 [89]	106 (11.3%)	Patients ≥65 years scheduled to undergo elective spine surgery, Japan	CIRS	CAM (twice daily)	x	x	x	
Villalpando-Berumen, 2003 [90]	667 (12%)	Medical patients >60 years old, Mexico	CIRS	CAM (daily)	x	x	x	x
Wang, 2019 [91]	323 (8.7%)	Patients undergoing laryngectomy for laryngeal cancer, China	CIRS (≥8)	CAM (once daily for first 6 days post-operatively)	x			
Elder, 2023 [92]	5886 (10%)	≥65 years presenting to the Emergency Department and admitted to family or internal medicine services, USA	Elixhauser Comorbidity Index (per 10 points)	CAM-ICU (twice daily)			x	x
Iterations of DSM used to measure delirium								
Koebrugge, 2010 [93]	107 (23.4%)	Vascular surgery, Netherlands	Number of diagnoses CCI	DOS then DSM – IV if DOS ≥ 3 (3 times per day)	x			
Lee, 2010 [94]	81 (13.6%)	Spinal surgery patients >70 years, Republic of Korea	Number of comorbidities (≥3)	CAM and DSM – IV (daily)	x			
Lima, 2010 [95]	199 (33.2%)	Geriatric inpatients >60 years old, Brazil	Number of diagnoses (≥5)	DSM – IV (daily)	x			
Schuurmans, 2003 [96]	92 (19.6%)	Hip fracture patients >70 years old, Netherlands	Number of comorbidities	DSM – IV (screened daily for first 6 days with DOSS)	x	x		
Srinonprasert, 2011 [97]	225 (48.9%)	Medical patients ≥70 years old, Thailand	Number of comorbidities (≥4)	DSM – IV (every 48 hrs)	x	x	x	
Zhang, 2019 [98]	825 (14.3%)	Hip fracture patients ≥65 years old, China	Number of comorbidities (≥2)	DSM 5	x	x	x	
Cerejeira, 2013 [99]	101 (36.6%)	≥60 year olds without dementia undergoing elective hip arthroplasty, Portugal	CCI	DSM-IV-TR criteria (screened with CAM daily for first 3 days post-operatively)	x			
Chu, 2016 [100]	544 (9.6%)	Elective and emergency orthopaedic patients≥60 years old, Taiwan	CCI	CAM confirmed by DSM – IV – TR (daily)	x	x	x	x
de Haan, 2023 [101]	2051 (16%)	>70 years undergoing hip fracture surgery, Netherlands	CCI	DSM 5	x	x	x	
Di Giorgio, 2022 [102]	214 (22%)	900-bed general hospital, Italy	CCI (without dementia)	DSM 5	x	x	x	
Franco, 2020 [103]	200 (25%)	Medical inpatients aged ≥60 years, Columbia	CCI-short form	DSM 5 (once)	x	x		
Huang, 2017 [104]	1016 (0.59%)	Total knee arthroplasty patients, Singapore	CCI	DSM-IV	x			
Ito, 2017 [105]	146 (19.9%)	Consecutive patients undergoing pancreaticoduodenectomy, Japan	Age adjusted CCI (excluding primary tumours for which surgery was performed)	DSM – IV	x	x	x	
Katipoglu, 2022 [106]	615 (27.6%)	Geriatric patients, Turkey	Deyo-Charlson	CAM-short form then DSM—IV or DSM 5	x			
Kroon, 2022 [107]	412 (19.9%)	Medical patients with COVID aged ≥60 years, Netherlands	CCI (continuous and grouped: 1–3, 4–6, >6)	DOSS then DSM 5 if DOS ≥ 3 (DOSS three times daily)	x	x [continuous]	x	
Lahariya, 2014 [108]	309 (26.2%)	Cardiac ICU, India	CCI	CAM-ICU then DSM-IV-TR (CAM-ICU daily)	x	x	x	x
Miao, 2018 [109]	112 (43.8%)	≥60 year olds undergoing elective open gastrointestinal tumour resection, China	CCI	DSM—IV (twice daily for first 7 days post-operatively)	x			
O’Keeffe, 1997 [110]	225 (41.8%)	Emergency admissions to geriatric ward, Ireland	CCI (excluding dementia)	DSM-3 (every 48 hours)	x			
Pendlebury, 2015 [111]	503 (20.1%)	Consecutive patients admitted to acute medical ward, UK	CCI (>3)	CAM – diagnosis confirmed using DSM-IV (daily)	x			
Pol, 2011 [112]	142 (7.0%)	Vascular surgery patients, Netherlands	CCI	DSM-IV-TR	x	x	x	
Pol, 2014 [113]	277 (5.8%)	Consecutive elective vascular surgery patients, Netherlands	CCI	DSM-IV-TR	x	x	x	
van der Sluis, 2017 [114]	436 (10.3%)	Elective or emergency operation for colorectal malignancy, Netherlands	CCI (0; 1–2; ≥3)	DOS ≥3 resulted in assessment using DSM-IV (DOS three times daily)	x			
Visser, 2015 [115]	463 (4.8%)	Elective vascular surgery patients ≥60 years, Netherlands	CCI	DOS ≥3 resulted in assessment using DSM-IV (DOS three times daily)	x	x	x	
Curyto, 2001 [116]	53 (22.6%)	Hospitalised residential and nursing home residents, USA	CIRS	DSM III R (within 48 hrs of admission and then every 5 days)	x			
Pasinska, 2018 [117]	750 (27.1%)	Stroke patients, Poland	CIRS	CAM then DSM5 (daily for 7 days)	x	x	x	x
Richardson, 2021 [118]	205 (40%)	Population-based cohort study of incident dementia, UK	CIRS-G	DSM-5 (daily)	x	x		
Wetterling, 2023 [119]	566 (46.6%)	Neuropsychiatric inpatients >65 years, Germany	CIRS (minus neuro-psychiatric item) Number of ICD-10 diagnoses	DSM – IV—TR (daily for first 7 days of admission)	x			
Iterations of ICD used to measure delirium								
Yang, 2023 [120]	115 147 (0.89%)	≥18 year olds undergoing shoulder arthroplasty, USA	Number of comorbidities (from list of 29)	ICD-9-CM	x	x	x	x
Abdullah, 2018 [121]	1330 020 (1.4%)	Admissions with Myocardial Infarction, USA	CCI	ICD-9-CM	x	x		
Bandini, 2020 [122]	3431 632 (0.96%)	Onco-surgical patients, Canada	CCI	ICD-9-CM			x	x
Bauernfreund, 2023 [123]	85 979 (1.6%)	Patients with severe mental illness, UK	CCI	ICD-10			x	x
Fineberg, 2013 [124]	578 457 (0.8%)	Population-based database of patients undergoing lumbar decompression and lumbar fusion surgery, USA	Modified CCI (myocardial infarction omitted; liver disease weighting altered)	ICD-9-CM codes	x	x		
Igwe, 2023 [125]	748 (15.5%)	All chronic kidney disease patients aged ≥65 years admitted to ICU, Australia	CCI (0; 1–2; ≥3)	ICD-10			x	x
Jones, 2019 [126]	2447 (12.9%)	Cardiac surgery, Australia	CCI	ICD-10	x	x	x	x
Lim, 2023 [127]	902 (39.1%)	≥75 years, geriatric medicine inpatients, Singapore	Age adjusted CCI	ICD-10	x	x	x	
Mohanty, 2022 [128]	34 713 (9.4%)	Surgical patients >50 years old, USA	CCI (mild, moderate, severe)	ICD-9 and ICD-10	x	x		
Pagali, 2023 [129]	4351 (12.4%)	Consecutive patients hospitalised for COVID-19 at 16 hospitals, USA	CCI	ICD-10-CM codes			x	x
Patil, 2020 [130]	42 980 (1.8%)	All patients ≥65 years undergoing primary percutaneous coronary intervention, USA	CCI	ICD-9-CM codes	x	x		
Quraishi, 2015 [131]	4508 (4.4%)	Hospitalised ≥18 year olds, USA	Deyo–Charlson (0–3; 4–6; ≥7)	ICD-9-CM codes			x	x
Yang, 2020 [132]	388 424 (0.9%)	Database of primary elective total hip replacements, USA	CCI—modified	ICD-9-CM	x	x		
Yang, 2022 [133]	1228 879 (1.0%)	Total knee replacement patients, USA	CCI—modified	ICD-9-CM	x	x		
Aziz, 2018 [134]	2006 522 (0.68%)	Hip replacement patients, USA	Elixhauser	ICD-9-CM	x	x		
Kassie, 2022 [135]	10 456 (25%)	Patients ≥65 years who had knee or hip surgery, Australia	Elixhauser	ICD-10-AM	x	x		
Ritchie, 2022 [136]	153 023 (49.9%)	Admissions with heart failure, USA	Elixhauser	ICD-9-CM	x	x		
4AT used to measure delirium								
Morandi, 2021 [137]	241 (16.2%)	COVID-19 patients ≥65 years old, Italy	Number of chronic diseases (excluding dementia)	4AT (on admission)	x	x		
Bellelli, 2016 [138]	1867 (22.9%)	Inpatients ≥65 years old, Italy	CCI	4AT (once)	x			
Demirtakan, 2024 [139]	236 (40.6%)	≥65 year olds attending ED with new-onset neurological and cognitive symptoms or worsening in baseline mental status, Turkey	CCI	4AT (on admission)	x	x		
Dogrul, 2020 [140]	108 (3.7%)	>65 year olds scheduled for elective general, orthopaedic and trauma surgery, Turkey	CCI	4AT (pre-operatively and post-operative days 3 and 7)	x			
Monacelli, 2022 [141]	1829 (22.9%)	Hospital inpatients ≥65 years old, Italy	CCI	4AT (once)	x	x	x	
McCullagh, 2023 [142]	95 (9.5%)	>65 year olds scheduled for major surgery, UK	Geriatric Index of Morbidity	4AT (daily on first four postoperative days)	x			
MDAS used to measure delirium								
Weckmann, 2012 [143]	51 (31.4%)	Inpatients admitted for bone marrow transplant, USA	HCT-CI	MDAS and DRS (2–3 times per week)	x			
DRS-R98 used to measure delirium								
O’Regan, 2018 [144]	191 (31.9%)	Medical inpatients ≥70 years old, Ireland	Modified CIRS-G	DRS-R98 (daily)	x	x	x	x

Abbreviations: CAM, Confusion Assessment Method; CAM-ICU, Confusion Assessment Method for the Intensive Care Unit; CCI, Charlson Comorbidity Index; USA, United States of America; TAVI—transcatheter aortic valve implantation; SAVR, surgical aortic valve replacement; CABG—coronary artery bypass graft; ICU, Intensive Care Unit; CIRS, Cumulative Illness Rating Scale; CIRS-G, Cumulative Illness Rating Scale, Geriatrics; DOS, Delirium Observation Screening Scale; DRS-R98, Delirium Rating Scale-revised-98.

### Interaction of MLTC with other risk factors for delirium

One study explored how MLTC may interact with other risk factors for delirium [145]. Despite showing no significant difference between CCI scores in those with and without post-operative delirium (POD) overall, this study demonstrated that patients with CCI score of zero were more likely to experience POD after receiving heavier sedation levels compared to light sedation levels whereas in patients with CCI > 0, the level of sedation was not related to incident POD.

### Impact of MLTC on delirium presentation

#### Delirium severity

Three studies examined the association between MLTC and delirium severity; two of these studies showed that greater burden of MLTC was associated with greater delirium severity. In a post-acute intensive care unit (ICU) in Germany, the number of medical comorbidities was found to predict delirium severity according to the CAM-ICU-7 [146]. MLTC measured using CCI was found not to be associated with delirium severity, measured using the Delirium Index, in care home residents admitted to hospital [147]. However, CCI was found to correlate with delirium severity when measured using the Memorial Delirium Assessment Scale (MDAS), in a study of older inpatients with infections, and this finding remained in multivariate analysis with IL-6 and sepsis [148].

#### Delirium motor subtype

Two studies explored whether MLTC was associated with the motor subtype of delirium. Both studies found that MLTC, measured using CIRS in stroke patients and CCI in long term adult inpatients, was not related to motor subtype of delirium [149, 150].

#### Delirium duration

Three studies explored the relationship between MLTC and duration of delirium. MLTC measured using the number of comorbid conditions was found to be independently associated with delirium duration in long stay geriatric medicine patients [151] and mixed medical/surgical patients [152]. MLTC measured using CCI was also found to be a significant independent risk factor for persistent delirium when using a cut point of ≥4 [153].

#### Delirium detection

Two studies explored the impact of MLTC on delirium recognition. When examining documentation of delirium symptoms in nursing notes of general medical patients, higher CCI scores were associated with an increased likelihood of documentation of delirium symptoms [154]. However, CCI-Short Form was not found to differ between hospital inpatients with delirium referred to a psychiatrist in whom the referrer recognised the delirium compared to those with unrecognised delirium [155].

### Impact of MLTC on treatment of delirium

There were no studies that examined the impact of MLTC on the treatment of delirium.

### The contribution of MLTC to outcomes following delirium

Six studies examined the impact of MLTC on outcomes following an episode of delirium. In cardiac ICU patients with delirium, CCI scores were higher in those who died than in those who survived [108]. CCI was also found to contribute to variation in cognitive decline following POD [156] and was associated with increased readmissions within 28 days of discharge from the Emergency Department with delirium without immediate hospitalisation [157]. However, CCI was found not to predict mortality in younger hospital inpatients with delirium [158]. The number of comorbid conditions was found to be associated with mortality in patients with delirium in long stay geriatric medicine wards [151]. MLTC measured using CIRS was found not to differ between those with and without poor recovery after delirium, defined by decline in Activities of Daily Living, new institutionalisation or death [159].

### The contribution of delirium to outcomes following hospitalisation in patients with MLTC

One study demonstrated that delirium was associated with increased 1-year mortality in patients with MLTC; 1-year mortality with delirium during most recent hospital admission was 54.5% compared to 34.8% in those without delirium [72].

### The contribution of delirium to the development of MLTC

There were no studies that explored whether delirium increased the risk of developing MLTC.

### Methods used to define delirium and multiple long-term conditions

In 140 reports included in this analysis, 137 reported using one measure of MLTC and 3 studies used two measures of MLTC. The most frequently used measure of MLTC was the CCI (*n* = 98, 70.0%), including two studies using a short form of the CCI (CCI-SF). Other measures used were a simple count of the number of comorbidities (*n* = 27, 19.3%), the CIRS (*n* = 12, 8.6%), and the Elixhauser comorbidity measure (*n* = 4, 2.9%). One study used the Haematopoietic Cell Transplantation-specific Comorbidity Index (HCT-CI) and one used the Geriatric Index of Morbidity.

Of 140 studies, 81 (57.9%) recorded delirium using versions of the CAM. Other methods of delirium ascertainment included various iterations of the Diagnostic and Statistical Manual of Mental Disorders (DSM) criteria in 32 (22.9%) reports, ICD criteria in 18 (12.9%) reports and the 4 A’s Test (4AT) in 6 (4.3%) reports. Two studies (1.4%) used the MDAS to determine presence of delirium and one study used the Delirium Rating Scale (DRS-R98).

## Discussion

This scoping review has shown that the majority of the literature on MLTC and delirium has evaluated whether MLTC is a risk factor for delirium. The few studies exploring the impact of MLTC on delirium presentation, duration or outcomes found conflicting results, while no studies explored how MLTC impacts on the treatment of delirium or whether having delirium increases the risk of developing MLTC. This scoping review has also highlighted methodological limitations in existing research, with numerous definitions used for both MLTC and delirium and heterogeneity of settings and populations studied.

### Heterogeneity in recording of MLTC

The majority of studies in our scoping review (98/140) used the CCI to identify MLTC. First published in 1987, the CCI contains 19 predefined comorbidities designed to predict 1-year mortality in hospitalised patients in longitudinal research studies [160]. It is widely used in research and a number of standardised methods for calculating CCI from ICD codes within large datasets exist, such as by Deyo et al. [161]. The CCI is weighted to adjust for the differing mortality risks seen with the included conditions and liver disease, diabetes and neoplasm have two different weights based on disease severity.

However, there are several limitations associated with the CCI. First, it contains a limited, pre-defined list of conditions that may not capture the true extent of MLTC. Second, it was designed to predict mortality, which though important, may not reflect other outcomes of importance to some patients such as treatment burden or quality of life. Third, healthcare has evolved over the past three decades and the weightings are unlikely to reflect the current clinical consequences of some of the included conditions. The starkest example of this is seen with HIV/AIDS, originally assigned a top weighting of 6 points, which has seen huge improvements in life expectancy in most high-income countries.

A revised version of the CCI was published in 1994, which included the original 19 comorbidities plus one point for each additional decade above 40 years of age [162]. It was not always clear which version of the CCI was being used by studies in our review. In multivariate analysis, age and CCI were frequently both included which may result in competing risks if the age adjusted CCI was used and may account for the number of studies which demonstrated CCI as a risk factor for delirium only in univariate analysis. This was demonstrated by Romanauski et al. who performed multivariate analysis with and without age and found that with age included, CCI did not independently predict delirium but when age was removed, CCI was an independent predictor of delirium [81].

Despite being a standardised measure, CCI was operationalised in studies included in our review in a number of ways. For example, some studies included CCI as a continuous variable within analysis while others created different cut-offs. This variability was shown to impact on results in two studies, both of which found that CCI was not significantly different between delirium and no delirium groups when analysed as a categorical variable but did become significantly different when entered as a continuous variable [78, 107]. Some studies omitted certain diseases if they were very common in the population being studied, e.g. dementia [110], making meta-analysis of scores across studies very challenging.

The agreed definition of MLTC is the co-existence of two or more chronic conditions, each one of which is either a physical non-communicable disease, a mental health condition or an infectious disease, all of long duration [1]. Even in the 27 studies which used a count of conditions in our scoping review, the cut point used was not consistently two or more and it was often unclear how the list of conditions was compiled or which conditions were included. Two studies measured MLTC using both disease counts and CCI. In aortoiliac surgery patients, neither was found to differ between patients with and without delirium [93]. However, in older patients undergoing elective surgery, both measures differed between delirium and no delirium groups in univariate analysis but only MLTC measured using disease counts was predictive of POD in multivariate analysis [43]. A further study of older neuropsychiatric inpatients found that neither CIRS nor number of ICD-10 diagnoses differed significantly between those with and without delirium superimposed on dementia [119].

### Heterogeneity in recording of delirium

There was also heterogeneity in how delirium was evaluated. Most studies used CAM and DSM criteria, but few stated exactly how these criteria were operationalised and previous work has shown that differing iterations and variable interpretation of criteria all significantly impact on diagnostic rates [163, 164]. Authors were transparent in most cases about which ICD codes had been included but these codes varied between studies. Delirium occurrence tended to be lower in studies using ICD codes to define delirium and this likely reflects the poor recognition and documentation of delirium by clinical teams [165]. The measures of delirium were also applied at different time points, with some studies being cross-sectional in nature, taking a snapshot of delirium at one time point; others were longitudinal, measuring delirium anywhere up to three times daily for entire admissions. This is relevant given the fluctuating nature of delirium and the high risk of missing delirium when participants were seen on only one or a few occasions during a hospital admission. Other studies included only incident delirium, developing during admission, and excluded prevalent delirium, which was present on admission. This methodological heterogeneity makes comparison across studies very challenging.

### Heterogeneity in setting

Over half (76/140) of the studies included in our scoping review sampled surgical patients. We know that the prevalence of delirium varies greatly by setting, with high rates in older people’s wards and after major emergency surgery [9], and may well account for some of the variability seen in our results. One setting that is relatively under-represented in our scoping review is the ICU, with only nine included studies based there despite the high prevalence of delirium seen on ICUs [9]. However, it may reflect our inclusion criteria not capturing the differing terminology used to describe delirium within this setting [166] and also the extensive use in these settings of tools such as the Acute Physiology and Chronic Health Evaluation II score (APACHE) and the American Society of Anaesthesiologists physical status classification system (ASA), which are well validated in ICU populations for predicting mortality, but do not specifically measure MLTC [167, 168].

Although the number of reports included in this review was more than expected, and there was a reasonable spread of studies in terms of geographical locations, few were based within low and middle income countries. This limits the generalisability of the results and emphasises the urgent need for research into MLTC and delirium in these countries, as highlighted by the Academy of Medical Sciences report on MLTC [1].

None of the studies set out primarily to answer a question related to the relationship between MLTC and delirium and this was often drawn out of secondary analysis of existing datasets. This may in part be explained by the propensity to date for research into MLTC to be conducted within primary care settings, in contrast to delirium, which is largely seen in hospitals due to its acute and life-threatening nature.

### Limitations of this scoping review

This scoping review had a number of limitations. Due to the inclusion criteria, the full extent of the delirium literature related to intensive care populations was likely not captured. We included only adults, in line with Academy Medical Sciences report on MLTC and much of the current delirium literature [1]. We did not attempt meta-analysis due to the heterogeneity of studies and the scoping nature of the review. We did not perform any analysis of bias as the main aim of this scoping review was to summarise the available literature and identify knowledge gaps.

### Future directions

This scoping review has highlighted multiple gaps in the current literature that require attention. First, there is a pressing need to standardise the definitions of both delirium and MLTC to ensure comparability of studies and generalisability. Work on specifying the conditions which should contribute to definitions of MLTC has progressed in recent years, with work by Ho et al. and the ADMISSION collaborative aiming to standardise definitions used across studies [5, 169]. While a standardised methodology for delirium ascertainment remains challenging, optimising the assessment of delirium in research studies has been identified as an essential target to advance delirium practice and knowledge [170].

Second, using these standardised measures for MLTC and delirium, we need to confirm whether people living with MLTC are more at risk of delirium, and if so, which conditions or combinations of conditions are most strongly associated with delirium. One study included within our review explored the predictive value of individual conditions, as well as overall burden of comorbidity measured using the CCI, in a cross-sectional delirium study of 1829 hospitalised patients in Italy [141]. They found that dementia, cerebrovascular disease and hearing impairment were independently associated with delirium, in line with recent work evaluating risk factors for delirium [171]. However, further detailed analysis of this kind is needed in large, representative datasets. This will assist with risk stratifying patients for delirium, enable targeted delirium prevention interventions and also facilitate more informed discussions with patients and relatives. Gaining insights into how MLTC impacts on the current treatment of delirium may also generate hypotheses for future interventional trials aiming to prevent or treat delirium. Additionally, more detailed analysis of the specific patterns of MLTC most associated with delirium may provide insight into the currently elusive underlying pathophysiology of delirium, potentially paving the way for targeted treatments.

Third, we need to understand more about the outcomes following delirium in patients with MLTC and whether having delirium increases the risk of developing MLTC. We know that delirium increases risk of cognitive decline and dementia [118], but we do not know whether delirium increases the risk of other long-term conditions. Given that delirium is hypothesised to be due to inflammatory or immune dysregulation [9], it seems plausible that such a severe insult (and the knock-on consequences of longer hospital stay, immobility and opportunity for iatrogenic harm) may increase the risk and/or severity of other long-term conditions, even after the resolution of the delirium. Delirium interventions aimed at prevention have been shown to be effective in around a third of patients [172]. If delirium is shown to increase risk of MLTC, targeted delirium prevention could significantly reduce the global burden of MLTC.

## Conclusions

Existing literature largely evaluates MLTC as a risk factor for delirium and is limited by significant heterogeneity in defining both MLTC and delirium. No existing studies focus on the impact of MLTC on the treatment of delirium or the effect of delirium on the development of MLTC following hospitalisation. Robustly designed research studies to definitively address these key knowledge gaps are urgently needed in order to inform future interventional studies, guide the improved design of services and ultimately, transform the lives of people living with MLTC and delirium.

## Supplementary Material

aa-23-1053-File002_afae120
