# Supplementary material for: The interrelationship between multiple long-term conditions (MLTC) and delirium: a scoping review
Source: Age Ageing. 2024 Jul 4;53(7):afae120. doi: 10.1093/ageing/afae120 (PMC11223896; doi:10.1093/ageing/afae120)
Supplement: aa-23-1053-File002_afae120 [file aa-23-1053-file002_afae120.docx]

# **Manuscript title: The interrelationship between multiple long-term conditions (MLTC) and delirium: A scoping review**

# **Supplementary material**

# Contents

1. Search strategy
2. Data extraction proforma
3. Additional graphs
4. PRISMA-ScR Checklist
5. Table of all 140 included studies
6. Table of systematic reviews meeting inclusion criteria
7. References

# Search strategy

## Medline

1. deliri$.ti,ab.
2. (acute adj2 (confusion$ or "brain syndrome" or "brain failure" or "psycho-organic syndrome" or "organic psychosyndrome")).mp.
3. (terminal$ adj restless$).mp.
4. toxic confus$.mp.
5. delirium/
6. confusion/
7. or/1-6
8. *psychoses, alcoholic/ or *alcohol withdrawal delirium/
9. *substance withdrawal syndrome/
10. 8 or 9
11. 7 not 10
12. multimorbidit$.mp
13. multi-morbidit$.mp
14. comorbidit$.mp
15. co-morbidit$.mp
16. polymorbidit$.mp
17. poly-morbidit$.mp
18. multicondition$.mp
19. multi-condition$.mp
20. MLTC.mp
21. "multiple long term conditions".mp
22. ((multiple or coexisting or co-existing or concurrent or con-current or comorbid or co-morbid) adj2 (disease$ or illness$ or condition$ or diagnos$ or morbid$)).mp
23. multimorbidity/
24. comorbidity/
25. Or/12-24
26. 11 AND 25

- Searched 16/11/2023
- 1628 results

## Embase

1. delirium/
2. (acute adj2 (confusion$ or "brain syndrome" or "brain failure" or "psycho-organic syndrome" or "organic psychosyndrome")).mp.
3. deliri$.ti,ab.
4. (terminal$ adj restless$).mp.
5. toxic confus$.mp.
6. or/1-5
7. *alcohol psychosis/
8. *delirium tremens/
9. *withdrawal syndrome/
10. 7 or 8 or 9
11. 6 not 10
12. multimorbidit$.ti,ab
13. multi-morbidit$.mp
14. comorbidit$.mp
15. co-morbidit$.mp
16. polymorbidit$.mp
17. poly-morbidit$.mp
18. multicondition$.mp
19. multi-condition$.mp
20. MLTC.mp
21. "multiple long term conditions".mp
22. ((multiple or coexisting or co-existing or concurrent or con-current or comorbid or co-morbid) adj2 (disease$ or illness$ or condition$ or diagnos$ or morbid$)).ti,ab
23. comorbidity/
24. multimorbidity/
25. Or/12-24
26. 11 AND 25
27. limit 26 to english language

- Searched 16/11/2023
- 5211 results

## Psycinfo (OVID)

1. exp delirium/
2. deliri$.ti,ab.
3. (acute adj2 (confusion* or "brain syndrome" or "brain failure" or "psycho-organic syndrome" or "organic psychosyndrome")).ti,ab,sh.
4. (terminal* adj restless*).ti,ab,sh.
5. toxic confus*.ti,ab,sh.
6. or/1-5
7. *drug withdrawal/
8. *alcohol withdrawal/
9. *alcoholic psychosis/
10. *delirium tremens/
11. or/7-10
12. 6 not 11
13. multimorbidit$.mp
14. multi-morbidit$.mp
15. comorbidit$.mp
16. co-morbidit$.mp
17. polymorbidit$.mp
18. poly-morbidit$.mp
19. multicondition$.mp
20. multi-condition$.mp
21. MLTC.mp
22. "multiple long term conditions".mp
23. ((multiple or coexisting or co-existing or concurrent or con-current or comorbid or co-morbid) adj2 (disease$ or illness$ or condition$ or diagnos$ or morbid$)).mp
24. comorbidity/
25. Or/13-24
26. 12 AND 25

- Searched 16/11/2023
- 729 results

## CINAHL

S1: toxic n1 confus* OR terminal* n1 restless* OR acute n2 "organic psycho?syndrome" OR acute n2 "psycho-organic syndrome" OR acute n2 "brain failure" OR acute n2 "brain syndrome” OR acute n2 confusion* OR deliri* OR mh "delirium management (iowa nic)" OR mh "confusion" OR mh "delirium"

S2: mh "psychoses, substance-induced+" OR mh "alcohol withdrawal delirium" OR mh "substance withdrawal syndrome"

S3: S1 OR S2

S4: MM (multimorbidit* or multi-morbidit* or comorbidit* or co-morbidit* or polymorbidit* or poly-morbidit* or multicondition* or multicondition* or “multiple chronic condition*” or “morbidity burden” or ((multiple or coexisting or co-existing or concurrent or con-current or comorbid or co-morbid) N2 (disease* or illness* or condition* or diagnos* or morbid*)))

S5: S3 AND S4

- Searched 16/11/2023
- 56 results

## Pubmed

((delirium) OR (acute confusion) OR (confusion)) AND ((comorbidity) OR (multimorbidit*) OR (multi-morbidit*) OR (co-morbidit*) OR (poly-morbidit*) OR (comorbidit*) OR (polymorbidit*) OR (multicondition*) OR (multi-condition*) OR (MLTC) OR ("multiple long term conditions"))

- Searched 16/11/2023
- 3171 results

# Data extraction proforma

| Study author and year of publication |  |
| --- | --- |
| Extracted by |  |
| Checked by |  |

| Setting, including country |  |
| --- | --- |
| Funding |  |

Population:

| Sample size |  |
| --- | --- |
| Sample size with delirium |  |
| Mean age (SD) |  |
| Sex (%female) |  |

Methods:

| Delirium diagnostic method used |  |
| --- | --- |
| MLTC diagnostic method used |  |
| Brief summary of study design |  |
| Main objectives of the study |  |

Results:

| *Outline key findings (within framework of original objectives for scoping review)* | |
| --- | --- |
| The impact of MLTC on delirium prevalence and incidence: |  |
| The impact of MLTC on delirium presentation: |  |
| The impact of MLTC on treatment of delirium in hospitalised patients: |  |
| The contribution of both delirium and MLTC to outcomes: |  |
| Other finding(s) or comments: |  |

# Additional graphs

Figure 1(a): Graph representing the proportion of studies exploring MLTC as a risk factor for delirium (n=125) using each method of recording MLTC divided into whether the results showed significance or non-significance between delirium and no delirium groups in univariate and multivariate analysis. The same studies may appear more than once as studies may have tested the relationship between MLTC and delirium in both univariate and multivariate analysis. The three studies using 2 methods to record MLTC were excluded from this analysis. “Other” includes Hematopoietic Cell Transplantation-specific Comorbidity Index and Geriatric Index of Morbidity. Abbreviations: CCI=Charlson Comorbidity Index; CIRS=Cumulative Illness Rating Scale.

Figure 1(b): Graph representing the proportion of studies exploring MLTC as a risk factor for delirium (n=125) using each method of recording delirium divided into whether the results showed significance or non-significance between delirium and no delirium groups in univariate and multivariate analysis. The same studies may appear more than once as studies may have tested the relationship between MLTC and delirium in both univariate and multivariate analysis. Abbreviations: CAM=Confusion Assessment Method; DRS-R98=Delirium Rating Scale-Revised 98, DSM=Diagnostic and Statistical Manual; ICD=International Code of Diseases; MDAS=Memorial Delirium Assessment Scale; 4AT=4 A’s Test.

Figure 1(c): Graph representing the proportion of studies exploring MLTC as a risk factor for delirium (n=125) carried out in each setting divided into whether the results showed significance or non-significance between delirium and no delirium groups in univariate and multivariate analysis. The same studies may appear more than once as studies may have tested the relationship between MLTC and delirium in both univariate and multivariate analysis. Abbreviations: ICU=intensive care unit

Figure 1(d): Graph representing the proportion of studies exploring MLTC as a risk factor for delirium (n=125) within each quartile of sample size divided into whether the results showed significance or non-significance between delirium and no delirium groups in univariate and multivariate analysis. The same studies may appear more than once as studies may have tested the relationship between MLTC and delirium in both univariate and multivariate analysis.

# PRISMA-ScR Checklist

**Preferred Reporting Items for Systematic reviews and Meta-Analyses extension for Scoping Reviews (PRISMA-ScR) Checklist**

| **SECTION** | **ITEM** | **PRISMA-ScR CHECKLIST ITEM** | **REPORTED ON PAGE #** |
| --- | --- | --- | --- |
| **TITLE** | | | |
| Title | 1 | Identify the report as a scoping review. | Title page |
| **ABSTRACT** | | | |
| Structured summary | 2 | Provide a structured summary that includes (as applicable): background, objectives, eligibility criteria, sources of evidence, charting methods, results, and conclusions that relate to the review questions and objectives. | 1 |
| **INTRODUCTION** | | | |
| Rationale | 3 | Describe the rationale for the review in the context of what is already known. Explain why the review questions/objectives lend themselves to a scoping review approach. | 2 |
| Objectives | 4 | Provide an explicit statement of the questions and objectives being addressed with reference to their key elements (e.g., population or participants, concepts, and context) or other relevant key elements used to conceptualize the review questions and/or objectives. | 2 |
| **METHODS** | | | |
| Protocol and registration | 5 | Indicate whether a review protocol exists; state if and where it can be accessed (e.g., a Web address); and if available, provide registration information, including the registration number. | 3 |
| Eligibility criteria | 6 | Specify characteristics of the sources of evidence used as eligibility criteria (e.g., years considered, language, and publication status), and provide a rationale. | 3 |
| Information sources* | 7 | Describe all information sources in the search (e.g., databases with dates of coverage and contact with authors to identify additional sources), as well as the date the most recent search was executed. | 3 |
| Search | 8 | Present the full electronic search strategy for at least 1 database, including any limits used, such that it could be repeated. | 3 |
| Selection of sources of evidence† | 9 | State the process for selecting sources of evidence (i.e., screening and eligibility) included in the scoping review. | 4 |
| Data charting process‡ | 10 | Describe the methods of charting data from the included sources of evidence (e.g., calibrated forms or forms that have been tested by the team before their use, and whether data charting was done independently or in duplicate) and any processes for obtaining and confirming data from investigators. | 4 |
| Data items | 11 | List and define all variables for which data were sought and any assumptions and simplifications made. | 4 and Supplementary material |

| Critical appraisal of individual sources of evidence§ | 12 | If done, provide a rationale for conducting a critical appraisal of included sources of evidence; describe the methods used and how this information was used in any data synthesis (if appropriate). | 4 |
| --- | --- | --- | --- |
| Synthesis of results | 13 | Describe the methods of handling and summarizing the data that were charted. | 4 |
| **RESULTS** | | | |
| Selection of sources of evidence | 14 | Give numbers of sources of evidence screened, assessed for eligibility, and included in the review, with reasons for exclusions at each stage, ideally using a flow diagram. | 4, 5 and figure 1, page 21 |
| Characteristics of sources of evidence | 15 | For each source of evidence, present characteristics for which data were charted and provide the citations. | Table 1,  pages 23-30 |
| Critical appraisal within sources of evidence | 16 | If done, present data on critical appraisal of included sources of evidence (see item 12). | N/A |
| Results of individual sources of evidence | 17 | For each included source of evidence, present the relevant data that were charted that relate to the review questions and objectives. | 5-7 |
| Synthesis of results | 18 | Summarize and/or present the charting results as they relate to the review questions and objectives. | 5-7 and table 1 |
| **DISCUSSION** | | | |
| Summary of evidence | 19 | Summarize the main results (including an overview of concepts, themes, and types of evidence available), link to the review questions and objectives, and consider the relevance to key groups. | 7-11 |
| Limitations | 20 | Discuss the limitations of the scoping review process. | 10 |
| Conclusions | 21 | Provide a general interpretation of the results with respect to the review questions and objectives, as well as potential implications and/or next steps. | 11 |
| **FUNDING** | | | |
| Funding | 22 | Describe sources of funding for the included sources of evidence, as well as sources of funding for the scoping review. Describe the role of the funders of the scoping review. | Title page |

JBI = Joanna Briggs Institute; PRISMA-ScR = Preferred Reporting Items for Systematic reviews and Meta-Analyses extension for Scoping Reviews.

* Where *sources of evidence* (see second footnote) are compiled from, such as bibliographic databases, social media platforms, and Web sites.

† A more inclusive/heterogeneous term used to account for the different types of evidence or data sources (e.g., quantitative and/or qualitative research, expert opinion, and policy documents) that may be eligible in a scoping review as opposed to only studies. This is not to be confused with *information sources* (see first footnote).

‡ The frameworks by Arksey and O’Malley (6) and Levac and colleagues (7) and the JBI guidance (4, 5) refer to the process of data extraction in a scoping review as data charting*.*

§ The process of systematically examining research evidence to assess its validity, results, and relevance before using it to inform a decision. This term is used for items 12 and 19 instead of "risk of bias" (which is more applicable to systematic reviews of interventions) to include and acknowledge the various sources of evidence that may be used in a scoping review (e.g., quantitative and/or qualitative research, expert opinion, and policy document).

*From:* Tricco AC, Lillie E, Zarin W, O'Brien KK, Colquhoun H, Levac D, et al. PRISMA Extension for Scoping Reviews (PRISMAScR): Checklist and Explanation. Ann Intern Med. 2018;169:467–473. [doi: 10.7326/M18-0850](http://annals.org/aim/fullarticle/2700389/prisma-extension-scoping-reviews-prisma-scr-checklist-explanation).

# Table of all 140 included studies

| **Author, year** | **Sample size**  **(% with delirium)** | **Age**  **(mean (SD) unless stated otherwise)** | **Primary objective of the study** | **Setting** | **Measure of MLTC** | **Measure of delirium** | **Impact of MLTC on delirium prevalence** | **Impact of MLTC on delirium presentation** | **The contribution of both delirium and MLTC to outcomes** |
| --- | --- | --- | --- | --- | --- | --- | --- | --- | --- |
| Abdullah,  2018 [1] | 1,330,020 (1.4%) | 66.8 (14.4) | To examine the prevalence and impact of delirium | Admissions with Myocardial Infarction, USA | CCI | ICD | CCI significantly higher in those with delirium compared to those without. Multivariate analysis not performed. |  |  |
| Afonso,  2010 [2] | 112  (34%) | 66 (19-84)  *median (range)* | To determine modifiable and non-modifiable predictors of delirium | Cardiac surgery ICU,  USA | CCI | CAM  (every 12 hrs) | CCI was significantly associated with delirium. CCI not included in multivariate analysis. |  |  |
| Alamri,  2018 [3] | 147  (21.8%) | 60-74 years - 65.3%  ≥75 years –  34.7% | To determine prevalence of delirium and identify associated factors | Older medical patients,  Saudi Arabia | Number of comorbidities (0-2; 3-5) | CAM  (on admission) | Number of comorbidities significantly different between delirium and no delirium groups. Remained significant in multivariate analysis with age and employment status and other covariates not listed in manuscript. |  |  |
| Alvarez,  2023 [4] | 143  (9.1%) | 78.97 (3.87) | To determine whether patients undergoing occupational therapy intervention have a lower incidence of post-operative delirium compared to the group treated only with standard measures | ≥75 year undergoing elective major surgery,  Chile | CCI | CAM  (twice a day for 5 days or until discharge) | No significant difference in CCI score between delirium and no delirium groups |  |  |
| Ansaloni,  2010 [5] | 357  (13.2%) | Delirium:  80.7 (6.6)  No delirium:  75.8 (7.4) | To assess the incidence and severity of post-operative delirium and identify risk factors | General elective and emergency surgery patients,  Italy | CIRS  (≥8) | CAM  (on post-operative days 1,2,3,6) | CIRS significantly higher in post-operative delirium compared to no post-operative delirium. CIRS≥8 remained a significant predictor of post-operative delirium in multivariate analysis with age, nitrate-containing drugs, Short Portable Mental State Questionnaire, hospital anxiety and depression score, activities of daily living, glycaemia, emergency surgery, perioperative transfusions and opiate intake. |  |  |
| Arias,  2022 [6] | 547  (23%) | 76.6 (5.1) | To examine whether parental education was related to delirium incidence and severity | Major, non-cardiac surgery,  USA | CCI | CAM | No univariate analysis presented. CCI independently predicted delirium in multivariate analysis with paternal education, participant’s education, adult reading test, deprivation index, cognitive activity scale, cognitive performance. |  |  |
| Arinzon,  2011 [7] | 322  (34%) | 79.9 (6.3) | To investigate the incidence and characterise predictors associated with delirium and how they influence outcomes | ≥65 year olds in long-stay geriatric medical centre,  Israel | Number of comorbidities | CAM |  | Number of comorbid conditions was significantly associated with delirium duration in multivariate analysis with age, albumin, sex, heart failure, urea /creatinine ratio, infection, renal failure, stroke, blood urea, nitrogen, sepsis | Number of comorbid conditions was significantly associated with mortality in multivariate analysis with 15 other variables including general characteristics, laboratory data, comorbid diseases and aetiologies of delirium. |
| Aziz,  2018 [8] | 2,006,522  (0.68%) | Delirium:  75 (0.2)  No delirium:  65 (0.1) | To determine whether post-operative delirium is associated with inpatient complication rates | Hip replacement patients,  USA | Elixhauser | ICD | Significantly more comorbidities in patients with delirium versus no delirium. Individual comorbidities were used in multivariate analysis rather than overall Elixhauser score. |  |  |
| Bandini,  2020 [9] | 3,431,632 (0.96%) | 64 (56-73) | To identify risk factors for post-operative delirium | Onco-surgical patients,  Canada | CCI | ICD | Univariate analysis not presented but % of delirium patients with CCI≥2 was higher than non-delirium patients. In multivariate analysis with 35 other variables, CCI was an independent predictor of delirium. |  |  |
| Bauernfreund,  2023 [10] | 85,979  (1.6%) | 42 (30–58) *median (IQR)* | To describe incidence of delirium and risk factors | Patients with severe mental illness,  UK | CCI | ICD codes /HES data | Univariate data not presented. CCI predicted delirium independent of age and sex. Fully adjusted multivariate analysis did not include CCI due to concerns about causality. |  |  |
| Béland,  2021 [11] | 612  (11%) | Delirium  80.6 (8.8)  No delirium  76.4 (7.6) | To confirm predicting factors of developing delirium | Older emergency department patients, Canada | Age adjusted CCI | CAM  (twice daily) | No difference in CCI between delirium and no delirium groups |  |  |
| Bellelli,  2016 [12] | 1867  (22.9%) | 82 (7.5) | To assess the point prevalence of delirium | Inpatients,  Italy | CCI | 4AT  (once) | No difference in CCI between delirium and no delirium groups |  |  |
| Billig,  2022 [13] | 732  (13.5%) | 72 (8.62) | Association of delirium with risk factors including comorbidities | Emergency department patients,  Brazil | CCI  (divided into categories of survival) | CAM  (on admission) | CCI percentage chance of survival significantly different between delirium and no delirium groups. Did not remain significant in multivariate analysis with circulatory disease, genitourinary disease, respiratory disease and age. |  |  |
| Carrasco,  2014 [14] | 374  (6.68%) | 76.1 (7.1) | To develop a clinical model for incident delirium | General medical inpatients, Chile | CCI | CAM  (every 48 hrs) | No difference in CCI between delirium and no delirium groups  (excluded prevalent delirium) |  |  |
| Cerejeira, 2013 [15] | 101  (36.6%) | 73.04 (6.29) | To determine the response of plasma cortisol and IGF-1 following surgical trauma, and their relationship with post-operative delirium | ≥60 years without dementia undergoing elective hip arthroplasty, Portugal | CCI | DSM-IV-TR criteria | CCI was not significantly different between those with and without delirium. Multivariate analysis not performed. |  |  |
| Chu,  2016 [16] | 544  (9.6%) | 74.2 (7.9) | To evaluate the Mini-Nutritional Assessment short form as a predictor of post-operative delirium | Elective and emergency orthopaedic patients, Taiwan | CCI | CAM  (daily), confirmed by DSM | CCI significantly higher in delirium versus no delirium group. CCI significantly predicted post-operative delirium in multivariate analysis with age, sex, living status, admission route, hearing impairment, body mass index, sodium level, time to surgery, type of surgery, blood transfusion, Barthel Index, Instrumental Activities of Daily Living, mini-nutritional assessment-short form, mini-mental state examination and Geriatric Depression Scale – 15. |  |  |
| Cunningham,  2019 [17] | 282  (14%) | 74.2 (5.8) | To test the hypothesis that CSF biomarkers would predict post-operative delirium | Elective hip and knee patients,  Northern Ireland | CCI | CAM  (daily for 3/7 post-operatively) | CCI significantly different between delirium and no delirium groups. Remained a significant predictor of delirium in multivariate analysis age, ASA, education, intelligence quotient, alcohol consumption, mini-mental state examination, cerebrospinal fluid AB42, T-tau and P-tau, type of surgery, intravenous opioid |  |  |
| Curyto, 2001 [18] | 53  (22.6%) | 83.3  (SD not provided) | To explore whether patients with delirium were more likely to die than patients without and explore the impact of baseline characteristics on this association | Residential and nursing home residents recruited and then reviewed for delirium when hospitalised, USA | CIRS | DSM III R (within 48hrs of admission and then every 5 days) | CIRS did not differ significantly between delirium and no delirium groups. |  |  |
| Czyzycki,  2021 [19] | 698  (25.6%) | 73  (median) | To investigate whether motor subtypes of stroke-associated delirium differ in predisposing factors, clinical characteristics and outcomes | Stroke patients, Poland | CIRS | DSM 5 |  | CIRS score did not differ significantly between hyperactive and hypoactive delirium groups. |  |
| Dasgupta,  2015 [20] | 1235  (28.7%) | 84.5  (SD not provided) | To determine how often delirious patients have geriatric syndromes and whether these are associated with poor recovery (decline in Activities of Daily Living, newly institutionalised, deceased) | Medical inpatients ≥70 years old,  Canada | CIRS | CAM  (every two days) |  |  | No difference in CIRS between those with poor recovery and those with functional recovery at discharge or at three months after discharge. |
| de Haan, 2023 [21] | 2051  (16%) | 80 (10) | To identify risk factors for delirium after hip fracture surgery | >70 years undergoing hip fracture surgery, Netherlands | CCI | DSM 5 | CCI significantly higher in delirium versus no delirium group. Not significant in multivariate analysis with sex, age, dementia, Parkinson’s disease, ward, blood loss, pneumonia, urinary tract infection and wound infection. |  |  |
| Demirtakan, 2024 [22] | 236  (40.6%) | 78.41 (7.93) | To reveal and compare the clinical outcomes and aetiological factors of older patients with delirium | ≥65 year olds attending ED with new-onset neurological and cognitive symptoms or worsening in baseline mental status, Turkey | CCI | 4AT  (on admission) | CCI significantly higher in delirium group compared to no delirium or coma group. Multivariate analysis not performed. |  |  |
| Devore,  2017 [23] | 552  (22.8%) | 76.8 (5.2) | To demonstrate that pre-surgical factors can predict the rate of cognitive decline after post-operative delirium | ≥70 years elective major non-cardiac surgery,  USA | CCI  (0;1;≥2) | CAM  (daily) |  |  | Contributed to variation in long-term cognitive decline in older participants with post-operative delirium but was not alone significantly associated with risk of cognitive decline following post-operative delirium |
| Di Giorgio, 2022 [24] | 214  (22%) | 67.88 (15.05) | To evaluate the occurrence of delirium in a large cohort of adults hospitalised for COVID-19 and its risk factors | 900-bed general hospital, Italy | CCI  (without dementia) | DSM 5 | CCI score significantly associated with diagnosis of delirium. Did not remain significant in multivariate analysis with age, Glasgow Coma Scale, dementia, pre-admission use of psychotropic drugs, C-Reactive Protein and Neutrophils-to-Lymphocytes Ratio |  |  |
| Dogrul,  2020 [25] | 108  (3.7%) | 71 (65–84) | To investigate the influence of pre-operative Comprehensive Geriatric Assessment and frailty on post-operative delirium | Elective post-operative general, orthopaedic and traumatology patients, Turkey | CCI | 4AT  (pre-operatively and post-operative days 3 and 7) | No difference in CCI between delirium and no delirium groups |  |  |
| Dworkin,  2016 [26] | 76  (13.2%) | 71 (6) | To develop a clinical tool to stratify older adults undergoing surgery according to their risk of post-operative delirium | Elective surgical patients, USA | Charlson-Deyo | CAM | Higher comorbidities, as measured using Charlson-Deyo index, increased the odds of delirium. Multivariate analysis not performed. |  |  |
| Eide,  2015 [27] | 143  (53.1%) | 83.5 (2.7) | Determine the incidence of post-operative delirium and identify risk factors for its development | Octogenarians undergoing elective transcatheter aortic valve implantation or surgical aortic valve replacement,  Norway | CCI | CAM  (daily for 5 days post-operatively) | No difference in CCI between delirium and no delirium groups |  |  |
| Elder, 2023 [28] | 5,886  (10%) | 77 (69-83)  *median (IQR)* | To evaluate the association between incident delirium with emergency department length of stay, time in hallways, and number of non-clinical patient moves | ≥65 years presenting to the emergency department and admitted to family or internal medicine services, USA | Elixhauser Comorbidity Index  (per 10 points) | CAM-ICU (twice daily) | Elixhauser comorbidity index independently predicted delirium with age, sex, English language, dementia, total emergency department non-clinical bed moves, emergency department hallway time and emergency department length of stay. Univariate analysis not presented. |  |  |
| Eschweiler,  2021 [29] | 880  (23.6%) | 77.8 (4.9) | To estimate a delirium risk model | Surgical patients >70 years,  Germany | CCI  Number of diseases (<=4,5-8,>=9) | CAM  (daily for 7 days) | Mean CCI and number of diseases significantly higher in delirium versus no delirium. CCI did not remain significant in multivariate analysis but number of diseases did, independent of age, ASA, frailty, polypharmacy, baseline cognition, sensory impairment, cut-to-suture-time, renal failure, cardio-pulmonary bypass, surgery type |  |  |
| Esmaeeli,  2022 [30] | 556  (14%) | Delirium  85 (7)  No delirium  80 (8) | To investigate the relationship between pre-operative frailty and new-onset post-operative delirium | ≥65 years old orthopaedic  trauma patients,  USA | CCI | CAM | CCI score significantly higher in delirium versus no delirium group. Not significant predictor of post-operative delirium in multivariate analysis with age, sex, single or widowed, mini-cognitive score, FRAIL score, glomerular filtration rate, falls within last year, ICU admission |  |  |
| Ferré,  2022 [31] | 67  (54%) | 78 (71–86)  *median (IQR)* | To demonstrate an association between atropinic burden and occurrence of post-operative delirium in hip fractured patients | ≥65 year olds urgent surgery for hip fracture, France | CIRS-G | CAM  (3 times daily until post-operative day 7 or until  Discharge) | No significant difference in CIRS-G score between delirium and no delirium |  |  |
| Fick, 2000 [32] | 20  (60%) | 80.6  (SD not provided) | To describe the recognition and management of delirium in hospitalised patients with and without dementia | Inpatients >65 years, USA | CCI | CAM  (daily) | CCI was significantly higher in delirious versus non-delirious patients. Multivariate analysis not performed. |  |  |
| Fineberg, 2013 [33] | 578,457 (0.8%) | Delirium 70  No delirium 55  (SD not provided) | To characterize the incidence, hospital costs, mortality, and risk factors associated with post-operative delirium after lumbar surgery | Population-based database from 2002–2009 of patients undergoing lumbar decompression  and lumbar fusion surgery, USA | Modified CCI (myocardial infarction omitted; liver disease weighting altered) | ICD-9-CM codes | CCI was significantly higher in delirium versus no delirium groups. CCI not included in multivariate analysis. |  |  |
| Flaherty,  2010 [34] | 148  (29.7%) | Delirium  85.3 (5.7)  No delirium 83.2 (7.1) | To compare outcomes in delirious versus non–delirious patients on a ward with a delirium room | Acute older persons ward, USA | CCI | Modified CAM (daily for 6 days) | No difference in CCI between delirium and no delirium groups |  |  |
| Fortes-Filho,  2016 [35] | 147  (41.5%) | 80 (9.1) | To evaluate the properties of the 10-point Cognitive Screener, a 2-min bedside tool, for predicting delirium in older adults with hip fracture | Hip fracture patients, ≥60 years,  Brazil | CCI  (0; 1; 2; ≥3) | CAM  (daily) | CCI did not differ significantly between delirium and no delirium group |  |  |
| Franco,  2020 [36] | 200  (25%) | Delirium  80.9 (7.7)  No delirium 73.4 (8.2) | To evaluate whether the Delirium Diagnostic Tool-Provisional differentiates sub-syndromal delirium from delirium and no delirium. | Medical inpatients, Columbia | CCI-Short Form | DSM 5  (once) | CCI-Short Form significantly higher in those with delirium compared to those without. Multivariate analysis was not performed. |  |  |
| Franz,  2023 [37] | 5042  (37.1%) | 61 (49-70) | To assess the extent of change in sedative medication use and to determine whether alterations to routine sedative medication use in a medical ICU leads to reduction in delirium rates | All adult (≥18 years old) patients admitted to the medical ICU,  USA | CCI | CAM-ICU (twice daily) | CCI score did not independently predict delirium in multivariate analysis with ARDS, organ failure, alcohol withdrawal severity, medication use and restraint. No univariate analysis presented. |  |  |
| Freter,  2016 [38] | 283  (57.6% pre-surgery; 41.7% post-surgery) | 82.9 (8.9) | To document prevalence and risk factors for delirium in pre and post-operative patients | Hip fracture patients >65 years,  Canada | Number of comorbidities | CAM  (pre-operatively then post-operative days 1, 3, and 5) | Number of comorbidities was significantly higher in patients with delirium pre-operatively but the difference was not significant post-operatively. Multivariate analysis not performed. |  |  |
| Galanakis,  2001 [39] | 105  (23.8%) | 74.9 (8.3) | To determine incidence and risk factors for the development of post-operative delirium | Elective and emergency hip surgery,  Germany | CCI | CAM  (daily) | CCI identified as a statistically significant risk factor for delirium in univariate logistic analysis. This did not remain significant when adjusted for age and sex. |  |  |
| Giuseffi,  2017 [40] | 226  (20%) | TAVR patients: 79.9 (9.5)  SAVR patients: 66.1(13.5) | Compare the incidence, odds, and mortality implications of delirium between patients undergoing TAVR and SAVR | Transcatheter Aortic Valve Replacement (TAVR) or Surgical Aortic Valve Replacement (SAVR) patients,  USA | CCI | CAM-ICU (twice daily) | CCI did not independently predict delirium in multivariate analysis with age, preoperative ejection fraction, midazolarn and procedure type. No data presented of univariate analysis. |  |  |
| Goldenburg,  2006 [41] | 77  (48.1%) | 81.9 (7.5) | To identify characteristics that are risk factors for delirium after surgical repair of a fractured hip | Hip fracture patients,  USA | Number of comorbidities (expressed as a morbidity index) | CAM  (daily) | Greater mortality index predicted post-operative delirium in univariate logistic analysis. When dichotomised with a cut-point of > 3, morbidity index was not an independent predictor of delirium in multivariate analysis with age, haematocrit, albumin, MMSE, Set Test score, functional decline, dementia, residence, use of multiple medications, central nervous system medications and presence of abnormal laboratory values. |  |  |
| Grover,  2013 [42] | 331  (100%) | 46.2 (19.3) | To evaluate risk factors for delirium and factors associated with mortality | Medico-surgical and emergency wards,  India | CCI | DSM IV-TR |  |  | No significant difference in CCI score between those who died and those who survived. |
| Guenther, 2013 [43] | 215  (31%) | Delirium  73.3 (71.2–75.4)  No delirium  68.5 (67.0–70.0)  *median (IQR)* | To assess pre-, intra-, and post-operative delirium  risk factors | Consecutive patients ≥ 50  years scheduled for cardiac surgery,  Germany | CCI | CAM-ICU  (daily for first 7 days post-operatively) | CCI independently predicted delirium in univariate and multivariate analysis with age, MMSE, length of cardiopulmonary bypass and Severe Inflammatory Response Syndrome (SIRS). |  |  |
| Guo,  2019 [44] | 244  (24.6%) | Delirium  72.3 (5.0)  No delirium  71.5 (5.1) | To investigate surgery-induced metabolic changes via comparison of post-operative delirium patients with non-post-operative delirium patients | Hip fracture surgery patients aged 65 to 80 years,  China | CCI | CAM  (twice daily for first three post-operative days) | CCI was not significantly different between those with and without delirium. Multivariate analysis not performed. |  |  |
| Hamann, 2005 [45] | 100  (7%) | 71.9  (60-92)  *mean (range)* | To determine the incidence of, and predictors for, the acute confusional state (ACS) in older patients after urologic surgery | ≥60 year olds undergoing urologic surgery,  Germany | CCI  (cut-point >3) | CAM  (daily) | CCI score did not differ between those with and without delirium |  |  |
| Hu,  2022 [46] | 531  (23.5%) | 68  *median* | To create an automated score to help predict post-operative delirium | Surgical patients,  China | CCI | CAM | Univariate analysis not presented. CCI independently predicted delirium in multivariate analysis with age, intraoperative blood loss, anaesthesia duration, extubation time, ICU admission, MMSE and post-operative neutrophil to lymphocyte ratio. |  |  |
| Huang, 2017 [47] | 1016  (0.59%) | Delirium  78 (73.9–82.1)  No delirium  67 (58.6–75.4)  *median (IQR)* | To investigate both the incidence and risk factors of post-operative delirium | Total knee arthroplasty patients,  Singapore | CCI | DSM-IV | CCI was not significantly different between those with and without delirium. Multivariate analysis not performed. |  |  |
| Igwe,  2023 [48] | 748  (15.5%) | 77.1 (7.2) | To determine the association between malnutrition and delirium in older chronic kidney disease (CKD) patients admitted to intensive care units | All CKD patients aged 65 and over admitted to ICUs, Australia | CCI  (0; 1-2; ≥3) | ICD | CCI ≥3 independently predicted delirium in multivariate analysis with age, sex, dementia diagnosis and nutritional status. Univariate analysis not presented. |  |  |
| Inouye,  2007 [49] | 491  (11.8% had delirium at discharge) | 79.1 (6.1) | To develop and validate a predictive model for persistent delirium at hospital discharge | General medical unit patients ≥70 years,  USA | CCI | CAM  (on admission and discharge) |  | CCI≥4 independently associated with delirium persisting at discharge in multivariate analysis with dementia, vision impairment and Activities of Daily Living impairment and restraint use. |  |
| Ito, 2017 [50] | 146  (19.9%) | 67.74 (9.69)  *median (IQR)* | To investigate the incidence and risk factors for delirium after pancreaticoduodenectomy | Consecutive patients who underwent pancreaticoduodenectomy, Japan | Age adjusted CCI (excluding primary tumours for which surgery was performed) | DSM-IV | CCI was significantly higher in delirium compared to no delirium patients. CCI did not remain a significant predictor of delirium in multivariate analysis including all patients with age, ASA, hypertension and sepsis. However, when stratified by age >70 years, CCI was an independent predictor of delirium in multivariate analysis with age and albumin. |  |  |
| Janssen,  2019 [51] | 627  (10.2%) | Delirium  79 (74–84)  No delirium  76 (73–80)  *median (IQR)* | To identify risk factors for post-operative delirium | ≥70 years, elective abdominal surgery for colorectal cancer or abdominal aortic aneurysm,  Netherlands | CCI≥7 | CAM or DSM 5 “regularly” | CCI (expressed as median score and CCI≥7) significantly higher in delirium compared to no delirium groups. Did not remain a significant predictor of delirium in multivariate analysis with neurologic comorbidity, hearing impairment, physical dependency, type of surgery, daily alcohol consumption, diabetes, post-operative anaemia, pre-operative anaemia, malnourishment, visual impairment, hypertension, previous delirium, renal impairment, cognitive impairment, ASA ≥ 3, smoker, ICU admission, blood transfusion and diagnosis of colorectal cancer. |  |  |
| Jones,  2019 [52] | 2447  (12.9%) | 66 (IQR 57-74)  *median (IQR)* | To identify the independent predictors of delirium | Cardiac surgery,  Australia | CCI | ICD | CCI was significantly higher in those with delirium compared to without. CCI remained a significant predictor of delirium in multivariate analysis with age, sex, admission source, peripheral vascular disease, respiratory disease, atrial fibrillation, cardiopulmonary bypass time and type of surgery |  |  |
| Juliebø,  2009 [53] | 237  (21.1% pre-operatively;  36.4% post-operatively) | 84 (79–88)  *median (IQR)* | To evaluate risk factors for preoperative and post-operative delirium | ≥65 years old, two orthopaedic surgery departments,  Norway | CCI >1 | CAM  (daily for up to five days post-operatively or discharge) | CCI score did not differ significantly between delirium and no delirium groups pre- or post-operatively |  |  |
| Kang,  2021 [54] | 683  (18.3%) | No delirium  66.2 (0.5)  Mild - Moderate delirium 70.2 (1.0)  Severe delirium  72.8 (2.7) | To investigate association between ankle brachial pressure index and post-operative delirium | Vascular surgery for peripheral vascular disease,  Republic of Korea | CCI | CAM-ICU | CCI significantly higher in those with delirium compared to those without. This remained significant in multivariate analysis with sex, age, occupation, education, length of surgery, ankle/brachial index, Rutherford class (severity of lower limb ischaemia) and ASA score. |  |  |
| Kassie,  2022 [55] | 10456  (25%) | 88 (84–91)  *median (IQR)* | To examine the risk of individual central nervous system-acting medicines used pre-operatively on delirium after hip or knee surgery | Patients ≥65 years who had knee or hip surgery, Australia | Elixhauser Comorbidity Index | ICD | Elixhauser Comorbidity Index was significantly higher in delirium versus no delirium group. Only used as a confounder in further analysis. |  |  |
| Katipoglu,  2022 [56] | 615  (27.6%) | 78.1 (6.8) | To assess the prevalence of delirium in patients with moderate or severe dementia | Geriatric patients,  Turkey | Deyo-Charlson | DSM | No difference in Deyo-Charlson between delirium and no delirium groups |  |  |
| Kennedy,  2014 [57] | 676  (9%) | 77 (8) | To identify risk factors associated with delirium in emergency department | Emergency department patients >65 years,  USA | CCI | CAM  (once) | CCI significantly higher in those with delirium compared to without delirium. CCI was not included in multivariate analysis. |  |  |
| Khan,  2022 [58] | 408  (16.7%) | 75 (8.2) | To determine if there are variables already present within our electronic health record that could be used to identify delirium | Acute medical unit patients ≥65 years,  USA | CCI (continuous and >2) | 3D CAM | CCI (as a continuous and dichotomous variable with cut-point >2) significantly higher in those with delirium compared to those without. CCI did not appear to be included in multivariate analysis |  |  |
| Kim,  2023 [59] | 257  (20.2%) | Delirium  76 (73 - 79)  No delirium  74 (71 - 77)  *median (IQR)* | To explore the relationship  between pre-operative electroencephalography and post-operative delirium | >70 year olds undergoing spinal surgery,  Republic of Korea | CCI | CAM  (four times daily) | CCI significantly higher in delirium versus no delirium. This did not remain significant in multivariate analysis with age, median dominant frequency (MDF), Montreal Cognitive Assessment score and mini nutritional assessment. |  |  |
| Kimura,  2023 [60] | 106  (11.3%) | 71.6 (5.9) | To investigate the prevalence and predictors of post-operative delirium | Patients ≥65 years scheduled to undergo elective spine surgery, Japan | CIRS | CAM  (twice daily) | CIRS significantly higher in delirium compared to no delirium groups. This did not remain significant in multivariate analysis with age, number of medications, mini-Cog score, length of hospital stay, haemoglobin, albumin, blood urea nitrogen, creatine and CRP |  |  |
| Koebrugge,  2010 [61] | 107  (23.4%) | Delirium  73.4 (6.4)  No delirium  67.8 (9.7) | Determine incidence, risk factors and outcomes of post-operative delirium | Vascular surgery  (elective and emergency),  Netherlands | Number of diagnoses and CCI | DOS then DSM IV if DOS ≥3  (3 times per day) | No difference in CCI or number of diagnoses between delirium and no delirium groups |  |  |
| Korc-Grodzicki, 2015 [62] | 416  (19%) | 80 (75–98)  *median (range)* | To determine the association between geriatric assessment variables and the risk of developing post-operative delirium | Surgical patients with solid tumours aged ≥ 75 years,  USA | CCI  (≥3) | CAM  (daily) | CCI≥3 significantly more likely in those with delirium compared to those without delirium. CCI≥3 a significant predictor of delirium in multivariate analysis with dependence for Instrumental Activities of Daily Living and falls. |  |  |
| Kroon,  2022 [63] | 412  (19.9%) | 76 (68–82)  *median (IQR)* | To identify determinants of delirium | Medical patients with COVID,  Netherlands | CCI (continuous and groups: 1-3, 4-6, >6) | DOS then DSM 5 | CCI divided into categories did not differ significantly between delirium and no delirium groups. CCI as a continuous variable did differ significantly. Did not remain significant in multivariate analysis with age, previous delirium, history of memory problems, Activity of Daily Living dependency, fall in the last 6 month and Clinical Frailty Scale. |  |  |
| Kuswardhani,  2017 [64] | 60  (100%) | 68.0 (6.6) | To elaborate the risk factors related to delirium severity | Geriatric inpatients ≥60 years,  Indonesia | CCI  (age adjusted) | MDAS |  | CACI correlated with MDAS score. More comorbidities associated with more severe delirium. Remained significant in multivariate analysis with IL-6 and sepsis. |  |
| Lahariya,  2014 [65] | 309  (26.2%) | Delirium  61.7 (13.5)  No delirium  57.0 (12.7) | To evaluate prevalence, risk factors and outcomes associated with delirium | Cardiac ICU,  India | CCI | DSM-IV-TR | CCI was significantly higher in those with delirium compared to without. CCI remained a significant predictor of delirium in multivariate analysis with 41 other variables. |  | In those with delirium, CCI was an independent predictor of mortality in multivariate analysis with age, diabetes mellitus, opioid use, acute infection, cardiogenic shock, temperature disturbances, arterial pH, APACHE-II Score, Sequential Organ Failure Assessment Score, number of medications |
| Lai,  2022 [66] | 345  (5.5%) | 73 (65-99)  *median (range)* | To investigate incidence, risk factors and outcomes of post-operative delirium | ≥65 years elective surgery for gastrointestinal cancer, Taiwan | CCI  (≥3) (minus age and cancer) | CAM  (daily) | CCI ≥3 independently predicted post-operative delirium in univariate and multivariate analysis with age, sex, cancer type and stage and operative details |  |  |
| Large,  2013 [67] | 49  (29%) | Delirium  77.8 (73.5-83.5)  No delirium  73.1 (70.1-76.5)  *median (IQR)* | To identify the risk factors for the development of delirium | Radical cystectomy patients ≥65 years,  USA | CCI  (Age adjusted) | CAM  (post-operative days 1,2,3,5,7) | No difference in CCI between delirium and no delirium groups |  |  |
| Lee,  2011 [68] | 425  (35%) | No dementia  79.4 (6.9)  Dementia  83.1 (6.2) | To determine the utility of pre-operative assessment of dementia for post-operative delirium | Hip fracture surgery,  USA | Number of comorbidities | CAM  (daily) | Number of comorbidities significantly predicted delirium in those without dementia. Remained a significant predictor of delirium in multivariate analysis with age, sex, BMI, duration of surgery, time to surgery and dementia status. |  |  |
| Lee,  2010 [69] | 81  (13.6%) | 73.5 (70-85)  *mean (range)* | To examine the incidence, pre- and post-operative risk factors and clinical results of post-operative delirium after spine surgery | Spinal surgery patients >70 years,  Republic of Korea | Number of comorbidities (≥3) | CAM and DSM IV  (daily) | Number of comorbidities did not differ significantly between delirium and no delirium groups |  |  |
| Lee,  2023 [70] | 1,353  (5.8%) | 80 (42-95)  *mean (range)* | To determine peri-operative risk factors and clinical outcomes of post-operative delirium | Consecutive patients undergoing hip bipolar hemi-arthroplasty for displaced femoral neck fractures,  Taiwan | CCI  (≥6) | CAM | Significantly more people with delirium had a CCI≥6 compared to age and sex matched control group. Remained significant in multivariate analysis with sex, age, ASA scores, waiting time before surgery, operation duration, operative timing and pre-operative anaemia |  |  |
| Leung,  2015 [71] | 50  (14%) | 66 (11) | To describe pre-operative and post-operative sleep disruption and its relationship to post-operative delirium | Patients ≥ 40 years scheduled for major non-cardiac surgery,  USA | CCI | CAM | CCI did not differ significantly between delirium and no delirium group |  |  |
| Li,  2015 [72] | 38  (18.4%) | 62.4 (11.8) | To evaluate the course of incident delirium and sub-syndromal delirium, their risk factors, and impact on patients’ cognitive function | Non-delirious consecutive patients scheduled for elective coronary artery bypass graft surgery,  Taiwan | CCI | CAM  (daily for one week post-operatively) | CCI was significantly higher in delirium compared to sub-syndromal or no delirium patients. No multivariate analysis performed. |  |  |
| Lim,  2023 [73] | 902  (39.1%) | 85.3 (6.2) | To determine association of frailty measured using Hospital Frailty Risk Score with delirium | ≥75 years, geriatric medicine inpatients, Singapore | CCI  (age adjusted) | ICD | CCI significantly different when comparing delirium versus no delirium groups. Not significant in multivariate analysis with age, frailty, dementia, diabetes, hypertension, hyperlipidaemia, chronic kidney disease, intracranial haemorrhage, stroke, myocardial infarction, pneumonia, urinary tract infection, urinary retention, constipation, infection, dehydration, hyponatremia, fragility fracture |  |  |
| Lima,  2010 [74] | 199  (33.2%) | 77.9 | To determine the impact of delirium on post-discharge mortality | Geriatric inpatients,  Brazil | Number of diagnoses  (≥5) | DSM 4  (daily) | No difference in number of diagnoses between delirium and no delirium groups |  |  |
| Liu,  2022 [75] | 309  (16.8%) | Delirium  81 (75-85)  No delirium  79 (73-83) | To investigate associations between pre-operative glycaemic control and physical performance and post-operative delirium | Hip fracture patients >65 years, China | CCI  (age adjusted) | CAM  (twice daily for two days) | Significantly higher CCI scores in delirium compared to no delirium group. CCI was a significant predictor of and post-operative delirium in multivariate analysis with baseline MMSE, Type II diabetes, pre-operative blood glucose, and stair climbing. |  |  |
| Liu,  2022 [76] | 184  (19.6%) | 68 (64-72)  *median (IQR)* | To analyse CCI and other risk factors for post-operative delirium | Thoracic and abdominal surgery patients ≥60 years,  China | CCI  (age adjusted) | CAM  (twice daily for 3 days) | CCI significantly higher in delirium versus no delirium. Remained significant in multivariate analysis with age, ASA, body mass index, pre-operative MMSE, albumin, albumin to fibrinogen ratio, D-dimer, total cholesterol, and pain scores. |  |  |
| Lochanie, 2018 [77] | 30  (66.7%) | 52.5 (31.6) | To assess the incidence of and risk factors for delirium | Mechanically ventilated patients in surgical ICU, Sri Lanka | Number of comorbidities (≥2) | CAM-ICU  (daily) | Proportion with 2 or more comorbidities not significantly different between delirium and no delirium groups |  |  |
| Ma,  2018 [78] | 2780  (100%) | No hospitalisation within 24 hours 57.7 (21.9)  Hospitalisation within 24 hours 65.8 (19.2) | To explore whether immediate hospitalisation influences the readmission risk in patients with delirium | Emergency department patients,  Taiwan | CCI | ICD |  |  | Patients with delirium who were hospitalised immediately had higher CCI scores than those who were not hospitalised immediately. Those not hospitalised immediately had a higher risk of readmission, especially subjects with more severe comorbidities (CCI≥3) |
| Mangnall, 2011 [79] | 118  (35%) | 71.81 (10.19) | To determine the prevalence and predictors of post-operative delirium in older patients after major colorectal surgery. | ≥50 years, admitted for elective major colorectal surgery,  Australia | Number of comorbidities | CAM  (daily for first 3 days post-operatively) | Mean number of comorbid conditions did not vary significantly between those who did not develop delirium and those who developed delirium on day 1 or by day 3. |  |  |
| Marcantonio,  2000 [80] | 126  (41.3%) | 79 (8) | To evaluate the role of delirium in the natural history of functional recovery after hip fracture surgery | Hip fracture patients >65 years,  USA | CCI  (≥4) | CAM  (daily) | Participants with delirium were more likely to have CCI≥4. Multivariate analysis was not performed. |  |  |
| McAlpine,  2008 [81] | 103  (17.5%) | 72  (SD not provided) | To determine delirium incidence and if any pre-operative, intra-operative, or post-operative parameters are associated with the development of delirium | Gynaecological cancer patients ≥60 years,  Canada | CCI | CAM  (post-operative day 1) | CCI score significantly higher in delirium versus no delirium group. Did not seem to be included in multivariate analysis. |  |  |
| McCullagh,  2023 [82] | 95  (9.5%) | 73.4 (67.0-79.8) | To identify the specific pharmacological and perioperative factors that modify the risk of post-operative delirium | Over 65 and scheduled for major surgery at five general and tertiary hospitals in the northeast of England | Geriatric index of morbidity | 4AT  (daily on first four post-operative days) | No significant difference in comorbidities between delirium and no delirium |  |  |
| McCusker,  2004 [83] | 318  (69.8%) | 83.5  (SD not provided) | To assess an instrument for measuring delirium severity | Medical patients ≥65 years,  Canada | CCI | CAM  (once) | CCI scores higher in delirium and DSD groups compared to no delirium groups. Multivariate analysis was not performed. |  |  |
| Miao,  2018 [84] | 112  (43.8%) | Delirium  71.8 (6.6)  No delirium  68.3 (7.8) | To investigate the possible predicative factors for delirium after open abdominal surgery | ≥60 year olds undergoing elective open gastrointestinal tumour resection,  China | CCI | DSM‑IV  (twice daily for first 7 days post-operatively) | CCI did not differ significantly between delirium and no delirium group |  |  |
| Mohanty,  2022 [85] | 34,713  (9.4%) | 66  *median* | Determine the relationship between post-operative delirium and development of dementia | Surgical patients,  USA | CCI  (mild, moderate, severe) | ICD | Patients with delirium were more likely to have higher Charlson scores. No multivariate analysis performed. |  |  |
| Monacelli,  2022 [86] | 1829  (22.9%) | 81.8 (5.5) | To assess the point prevalence of delirium | Hospital inpatients,  Italy | CCI | 4AT  (once) | Delirium group had significantly higher CCI. Did not remain significant in multivariate analysis with age, cerebrovascular disease, dementia, chronic lung disease, hemiplegia, visual impairment and hearing impairment. |  |  |
| Morandi,  2021 [87] | 241  (16.2%) | 77.5 (65.6 - 85.0)  *median (IQR)* | To investigate the association between delirium duration and in-hospital mortality | COVID patients,  Italy | Number of chronic diseases | 4AT | Number of chronic diseases significantly higher in delirium versus non-delirium groups. Multivariate analysis was not performed. |  |  |
| Moreno-Gaviño,  2012 [88] | 1632  (11%) | 77.9 (9.8) | To assess the prevalence of delirium and associated risk factors, and their impact on survival, in polypathological patients | Outpatients and hospital at home patients,  Spain | CCI | CAM | High comorbidity load on CCI was predictive of delirium. Data was not presented in a table, only mentioned in the text. Multivariate analysis not performed. |  |  |
| Mosello,  2020 [89] | 497  (18%) | 79 (7) | To identify predictors of delirium | Cardiac ICU patients ≥65 years,  Italy | CCI | CAM-ICU  (daily) | CCI was significantly higher in those with delirium compared to without. CCI remained a significant predictor of delirium in multivariate analysis with age, dementia, modified rapid emergency medicine score, ST elevation myocardial infarction and respiratory failure. |  |  |
| Naksuk,  2017 [90] | 11,079  (8.3%) | Delirium  73.1 (13.9)  No delirium  66.6 (15.2) | To examine the effect of delirium and antipsychotic therapy among coronary care unit patients | Patients admitted to coronary care unit,  USA | CCI | CAM-ICU (twice daily) | CCI score significantly higher in delirium compared to no delirium group. CCI not included in multivariate analysis. |  |  |
| Narayanan,  2022 [91] | 50  (22%) | 69.5 (2.8) | To find the incidence of post-operative delirium | Post-operative cancer patients,  India | Number of comorbidities (≥3) | Short-CAM (twice daily for 3 days) | Number of comorbidities ≥3 showed a statistically significant association with post-operative delirium. No multivariate analysis performed. |  |  |
| Ng,  2019 [92] | 280  (12.9%) | 63.6 (13.7) | To determine the incidence and risk factors of delirium in a cohort of ischaemic stroke within the first week of stroke | Consecutive patients with acute ischaemic stroke  aged ≥ 18 years,  Australia | Number of comorbidities (≥2)  (from  pre-defined list of conditions) | CAM  (within 7 days of stroke) | Number of participants with two or more comorbidities did not differ significantly between delirium and no delirium group |  |  |
| O’Keefe,  1997 [93] | 225  (41.8%) | Delirium  82 (4)  No delirium  82 (6) | Determine whether delirium was a predictor of adverse outcome | Emergency admissions to geriatric ward,  Ireland | CCI  (excluding dementia) | DSM-3  (every 48 hours) | No difference in CCI between delirium and no delirium groups |  |  |
| O’Regan,  2018 [94] | 191  (31.9%) | 80 (10)  *median (IQR)* | To identify predictors of incident delirium development | Medical inpatients,  Ireland | modified-CIRS-G | DRS-R98  (daily) | Modified Cumulative Illness Rating Scale Score was significantly higher in those with delirium compared to without. Remained significant in multivariate analysis with sex, age, dementia, Barthel Index and hearing impairment. |  |  |
| Pagali,  2023 [95] | 4351  (12.4%) | 71.1 (16.8) | To evaluate the performance of natural language processing-delirium algorithm for detection of delirium and to determine characteristics associated with identification of delirium | Consecutive patients hospitalized for COVID-19 at 16 hospitals,  USA | CCI | ICD-10-CM coding | CCI independently predicted delirium in multivariate analysis with age, length of stay, sex and ethnicity. Univariate analysis not presented. |  |  |
| Pasinska,  2018 [96] | 750  (27.1%) | 71.8 (13.1) | To assess frequency of delirium and build a predictive model | Stroke patients,  Poland | CIRS  (total) | DSM5  (daily for 7 days) | CIRS, expressed as total score, severity index and comorbidity index, significantly different between delirium and no delirium. CIRS total score was also an independent risk factor for delirium in multivariate analysis with Montreal Cognitive Assessment, neglect, vision deficit, white blood cell count on admission, Modified Rankin Scale. |  |  |
| Patil, 2020 [97] | 42,980  (1.8%) | Delirium  76.0 (7.6)  No delirium  73.7 (7.0) | To assess the impacts of delirium in older patients undergoing percutaneous coronary intervention (PCI) following ST-elevation myocardial infarction (STEMI) | All patients ≥65 admitted with a primary diagnosis of STEMI undergoing PCI during a four year period, USA | CCI | ICD-9-CM codes | Compared with STEMI patients without delirium, those with delirium were more likely to have more underlying comorbidities. CCI included as a confounder only in further analysis. |  |  |
| Pendlebury,  2015 [98] | 503  (20%) | 72 (16–99)  *median (range)* | To determine age-specific rates of delirium and associated factors in acute medicine | Consecutive patients admitted to acute medical ward,  UK | CCI  (>3) | CAM then DSM-IV  (CAM daily) | In ≥65 year olds, CCI did not differ significantly between delirium and no delirium group |  |  |
| Pioli,  2019 [99] | 939  (31.1%) | Delirium  86.9 (5.1)  No delirium  84.9 (5.5) | To evaluate the association between delirium onset and time to surgery | Hip fracture patients ≥75 years,  Italy | CCI | CAM  (daily) | CCI score significantly higher in delirium versus no delirium group. Did not remain significant in multivariate analysis with age, living in nursing home, cognitive impairment, Lawton Index and time to surgery. |  |  |
| Pol,  2011 [100] | 142  (7.0%) | 68 (11) | To determine whether the Groningen Frailty Indicator has a positive predictive value for post-operative delirium | Vascular surgery patients, Netherlands | CCI  (0-19 points) | DSM-IV-TR | CCI score was significantly higher in delirium versus non-delirium group. CCI did not independently predict delirium in multivariate analysis with impaired renal function, elevated C-reactive Protein, high ASA score, Delirium Observation Screening scale score of >3  Points, ICU admission and hospital length of stay |  |  |
| Pol, 2014 [101] | 277  (6%) | 69 (11) | To analyse the relationship between C-reactive Protein and post-operative delirium | Consecutive elective vascular surgery patients, Netherlands | CCI | DSM IV TR | CCI significantly higher in those with compared to without delirium. CCI did not remain significant in multivariate analysis with age, sex and CRP. |  |  |
| Quraishi,  2015 [102] | 4508  (4.4%) | 59 (18) | To determine whether pre-hospital 25-hydroxyvitamin D levels are associated with the risk of hospital-acquired new-onset delirium | Hospitalised ≥18 year olds, USA | Deyo–Charlson Index  (0–3; 4–6; ≥7) | ICD 9 | Deyo-Charlson 4-6 and ≥7 independently associated with delirium in multivariate analysis with age, sex, race, medical versus surgical admission, Vitamin D level. Univariate analysis not performed. |  |  |
| Radinovic,  2015 [103] | 270  (53%) | 78.1 (8.3) | To evaluate factors contributing to delirium | Hip fracture patients ≥60 years, Serbia | CCI >1 (without age) | CAM  (multiple times, at least daily) | Participants with delirium were significantly more likely to have CCI>1 than those without delirium. This did not remain significant in multivariate analysis with age, vision impairment, Short Portable Mental Status Questionnaire, Global Depression Scale and ASA status. |  |  |
| Radinovic,  2019 [104] | 277  (53%) | Delirium  80.95 (7.12)  No delirium  74.67 (8.11) | To examine the impact of intraoperative blood pressure, blood pressure fluctuation, and pulse pressure on post-operative delirium | >60 years with  hip fracture,  Serbia | CCI | CAM  (daily for 7 days post-operatively) | CCI score significantly higher in delirium compared to no delirium group. CCI included only as a confounder in further multivariate analysis. |  |  |
| Ranhoff,  2006 [105] | 401  (29.2%) | 78.1 (8.8) | To identify predisposing and precipitating factors for delirium | ≥60 year olds in medical unit,  Italy | CCI (continuous) | CAM  (daily) | CCI score significantly higher in delirium versus no delirium group. Multivariate analysis not performed as once dichotomised at a cut point of >7, CCI was no long significantly different between delirium and no delirium groups. |  |  |
| Richardson, 2021 [106] | 205  (40%) | 82.0 (6.5) | To measure the effect of delirium on cognition, independent of illness severity | Population-based cohort study of incident dementia, UK | CIRS-G  (total score) | Standardised procedure  based on DSM-5 | CIRS-G differed significantly between delirium and no delirium. CIRS-G included only as a confounder in further multivariate analysis. |  |  |
| Ritchie,  2014 [107] | 710  (12.3%) | 83.1 (7.4) | Describe association between C-reactive Protein and delirium | Medical Admissions Unit patients >70 years, UK | CCI | CAM  (once within 72hrs of admission) | No difference in CCI between delirium and no delirium groups. |  |  |
| Ritchie,  2022 [108] | 153,023 (49.9%) | Delirium  80.2 (71.3-86.5)  No delirium  74.5 (62.9-82.8)  *median (IQR)* | To explore the prevalence and predictors of delirium and its impact on outcomes | Admissions with heart failure,  USA | Elixhauser | ICD | Elixhauser score was significantly higher in those with delirium compared to those without delirium. Multivariate analysis not performed. |  |  |
| Robinson, 2009 [109] | 144  (44%) | 64 (9) | To identify risk factors, and determine outcomes for the development of post-operative delirium | >50 year olds scheduled for an operation requiring a post-operative intensive care unit admission,  USA | CCI | CAM-ICU  (daily) | CCI was significantly higher in those with compared to without delirium. Text states that “The following predictors remained in the final multivariable logistic model: Mini-Cog test (cognitive dysfunction), Charlson Index (burden of comorbidities), and haematocrit” but this data is not presented. |  |  |
| Romanauski,  2018 [110] | 6338  (9.6%) | Delirium  67.4 (17.2)  No delirium  60.4 (17.0) | To analyse association between post-operative delirium and pre-operative variables | General anaesthetic patients admitted to ICU post-operatively following non-cardiac or non-intracranial surgery,  USA | CCI  (without age) | CAM-ICU | CCI was significantly higher in those with delirium compared to without. CCI remained a significant predictor of delirium in multivariate analysis with sex, body mass index, surgery duration, emergency operation, blood transfusion, vasopressor infusion, opioid, post-operative mechanical ventilation, APACHE III score. CCI was not a significant predictor of delirium when age was included in the multivariate analysis. |  |  |
| Rudberg,  1997 [111] | 432  (14.8%) | 75.2 (65-95)  *mean (range)* | To determine the natural history of delirium | Inpatient surgical and medical wards at a university hospital, USA | number of medical diagnoses | CAM then DSM-III-R |  | The group with delirium on multiple days had a  higher average number of diagnoses |  |
| Saljuqi,  2020 [112] | 163  (26%) | 71 (7) | To assess the impact of frailty on delirium and the impact of delirium on outcomes in emergency general surgery | ≥65 years undergoing emergency general surgery,  USA | Number of comorbidities (≥3) | CAM | Significantly more of the delirium group had ≥3 comorbidities compared to the no delirium group. This remained significant on multivariate analysis with age, sex, race, weight, and insurance coverage, vital parameters, laboratory parameters, ASA class, and diagnosis |  |  |
| Schuurmans,  2003 [113] | 92  (19.6%) | Delirium  82.6 (6.9)  No-delirium  82.2 (6.6) | To identify risk factors useful for early recognition of delirium by nurses | Hip fracture patients, Netherlands | Number of comorbidities | DSM – IV (DOSS used for screening daily for first 6 days) | Mean number of comorbid conditions was significantly higher in delirium versus no delirium. No multivariate analysis performed. |  |  |
| Sieber,  2018 [114] | 200  (36.5%) | 81.8 (7.7) | To determine whether limiting sedation levels during spinal anaesthesia reduces incident delirium overall | Non-elective hip fracture repair patients ≥65 years, USA | CCI | DSM-IV-TR | The adjusted hazard ratio (adjusted for age, MMSE, fracture type, Geriatric Depression Scale score) showed that patients with the least comorbidity (CCI=0) were twice as likely to develop delirium after receiving heavier sedation levels than after receiving lighter sedation levels. In patients with higher baseline comorbidity, as indicated by a CCI score of more than 0, the level of sedation was not related to delirium. |  |  |
| Singler,  2014 [115] | 133  (14.3%) | 83.4 (5.5) | To assess the prevalence of delirium and identify delirium-associated patient characteristics | Emergency Department patients,  Germany | CCI  (≥3) | Short-CAM | CCI ≥3 not associated with delirium |  |  |
| Smith,  2015 [116] | 63  (37%) | 52.7 (16.8) | To determine if impaired pre-transplant cognitive functioning would be associated with greater risk of post-operative delirium | Lung transplant patients, USA | CCI | CAM  (daily) | CCI not associated with delirium |  |  |
| Son,  2022 [117] | 33  (51.5%) | 72.6 (13.1) | To develop accurate and explainable machine learning models for three psychomotor behaviours of delirium for hospitalized adults | Adult inpatients at one long term care hospital, Republic of Korea | CCI | Short-CAM |  | CCI did not differ significantly between different cases of motor subtypes of delirium |  |
| Srinonprasert,  2011 [118] | 225  (48.9%) | 78 (70-97)  *mean (range)* | To determine risk factors associated with delirium | Medical patients,  Thailand | Number of comorbidities (≥4) | DSM  (every 48 hours) | Delirium group significantly more likely to have ≥4 comorbid illnesses. Did not remain significant in multivariate analysis with sex, age, uraemia, hyponatremia, presence of infection, severe illness, dementia, depression, impaired Basic Activities of Daily Living. |  |  |
| Sugi,  2023 [119] | 158  (33.5%) | 79 (75–91)  *median (range)* | To investigate the risk factors for post-operative delirium | Patients ≥75 years old undergoing elective surgery for gastro-enterological cancer, Japan | CCI  (≥8) | CAM  (three times daily) | CCI significantly higher in delirium versus no delirium group. This difference did not remain in multivariate analysis with age, Vulnerable Elders Survey-13, Short Physical Performance Battery, Mini Nutritional Assessment Short-Form, MMSE and regular use of benzodiazepine. |  |  |
| Tan,  2008 [120] | 53  (23%) | Delirium  66.8 (7.5)  No delirium  61.5 (8.8) | To determine the incidence and predictors of delirium after cardiac surgery. | Elective cardiac surgery patients,  USA | CCI | CAM  (daily for first 7 days post-operatively) | CCI was significantly higher in people with delirium compared to no delirium. Multivariate analysis not performed. |  |  |
| Tkacheva,  2017 [121] | 181  (7.2%) | 77.3 (7.9) | To evaluate the effect of a short training course on the identification of delirium | Acute hospital,  Russia | CCI | CAM-ICU (once) | CCI score significantly higher in delirium versus no delirium group. Multivariate analysis was not performed. |  |  |
| Tognoni,  2011 [122] | 90  (8.8%) | 74.3 (0.40) | To investigate the occurrence of post-operative delirium in  older patients undergoing urological surgery and to identify risk factors | Consecutive patients undergoing urological surgery,  Italy | Number of comorbidities (≥2) | CAM  (daily for 7 days post-operatively) | Patients with delirium did not differ significantly to those without delirium in terms of number of comorbidities ≥2. |  |  |
| van der Sluis,  2017 [123] | 436  (10.3%) | 67 (40–90)  *median (range)* | To identify risk factors for post-operative delirium after colorectal operation for malignancy | Elective or emergency operation for colorectal malignancy,  Netherlands | CCI  (0; 1-2; ≥3) | DSM-IV  (if screening DOS ≥3)  . | CCI not a significant predictor of post-operative delirium in univariate analysis |  |  |
| Villalpando-Berumen,  2003 [124] | 667  (12%) | Delirium  75.8 (6.7)  No delirium  71.3 (8.1) | To determine incidence, risk factors and outcomes for delirium | Medical patients,  Mexico | CIRS | CAM  (daily) | CIRS significantly higher in delirium versus no delirium. Remained a significant predictor of delirium in multivariate analysis with age, length of stay, leukocytes, haematocrit and albumin. |  |  |
| Visser,  2015 [125] | 463  (4.8%) | 72 (66-77)  *median (IQR)* | To determine the incidence of and specific pre-operative and intra-operative risk factors for post-operative delirium | Elective vascular surgery patients,  Netherlands | CCI | DSM IV  (if screening DOS >3) | CCI significantly higher in those who did develop delirium. Did not remain significant in multivariate analysis with current smoking, increased comorbidity, hypertension, diabetes mellitus, preoperative cognitive impairment, open aortic surgery or amputation surgery, elevated CRP level, blood loss and age ≥80. |  |  |
| Voyer,  2007 [126] | 104  (68.3%) | 85.9 (5.7) | To identify factors associated with delirium severity | Geriatric and medical patients ≥65 years,  Canada | CCI | CAM |  | CCI did not differ significantly between mild delirium and moderate to severe delirium. |  |
| Voyer,  2008 [127] | 226  (100%) | 82.4 (7.3) | To evaluate documentation of nurse-reported delirium symptoms | General medical patients ≥65 year olds,  Canada | CCI | CAM |  | Higher level of comorbidity was associated with documentation of delirium symptoms in nursing notes. |  |
| Wang,  2017 [128] | 265  (18.5%) | Delirium  73.6 (5.9)  No delirium  69.6 (4.7) | To evaluate the incidence of delirium after total knee arthroplasty and to identify specific risk factors | ≥65 years undergoing elective total knee arthroplasty,  Republic of Korea | CCI  (2 or more groups) | CAM  (triggered by concerns about orientation) | CCI did not significantly predict post-operative delirium |  |  |
| Wang,  2019 [129] | 323  (8.7%) | Post-operative delirium  65.5 (6.4)  No post-operative delirium  61.7 (8.8) | To explore the risk factors and incidence of post-operative delirium | Patients undergoing laryngectomy for laryngeal cancer,  China | Cumulative Illness Rating Scale  (≥8) | CAM  (once daily for first 6 days post-operatively) | Mean CIRS score and CIRS ≥8 did not differ significantly between post-operative delirium and no post-operative delirium groups |  |  |
| Weckmann,  2012 [130] | 51  (31%) | 53.3 (10.7) | Describe pre-and post-stem cell transplant risk factors for delirium | Inpatients admitted for bone marrow transplant, USA | HCT-CI | MDAS and DRS  (2-3 times per week) | No difference in comorbidity score between delirium and no delirium groups |  |  |
| Wetterling, 2023 [131] | 566  (46.6%) | 79.5 (7.7) | To explore the impact of different types of brain pathology on rates of delirium superimposed on dementia | Neuropsychiatric inpatients >65 years,  Germany | CIRS  (minus neuro-psychiatric item),  number of comorbidities | DSM IV TR  (daily for first 7 days of admission) | CIRS (minus neuro-psychiatric item) did not differ between groups (delirium, delirium superimposed on dementia, dementia, other). CIRS and number of comorbidities did not differ between delirium and no delirium groups in those with dementia. |  |  |
| Wintermann,  2020 [132] | 267  (4.1%) | 61.6 (25.6–71.9)  *mean (range)* | To assess the delirium severity, its risk factors and association with adverse patient outcomes | Post-acute intensive care units,  Germany | Number of comorbidities | CAM-ICU |  | Number of medical comorbidities was significantly associated with delirium severity in univariate analysis. No multivariate analysis performed. |  |
| Witlox,  2011 [133] | 76  (39.5%) | Delirium  84.7 (5.1)  No delirium  82.4 (4.6) | To examine if baseline cerebrospinal fluid biomarkers are associated with greater risk of delirium | Hip fracture patients, Netherlands | Number of comorbidities | CAM | Number of comorbidities did not differ between delirium and no delirium groups |  |  |
| Xue,  2016 [134] | 358  (7.8%) | Delirium  78.14 (5.33)  No delirium  74.84 (6.39) | To investigate the occurrence of post-operative delirium  in older patients undergoing transurethral resection of prostate (TURP) and to identify  risk factors | ≥65 years and undergoing  TURP,  China | Number of comorbidities  (≥2) | CAM  (daily for 7 days post-operatively or if signs of confusion) | Patients who developed post-operative delirium had significantly more comorbidity. Did not remain significant in multivariate analysis with pain and age. |  |  |
| Yang,  2020 [135] | 388,424 (0.9%) | Delirium  77 (69-83)  No delirium  65 (57-74) | To investigate the incidence and risk factors associated with delirium | Database of primary elective total hip replacements,  USA | CCI  (modified) | ICD | Significantly higher CCI scores in those with post-operative delirium compared to those without. CCI not included in multivariate analysis. |  |  |
| Yang,  2022 [136] | 1,228,879 (1.0%) | Delirium  75 (69–81)  No delirium  66 (59–73)  *median (IQR)* | To investigate the incidence and risk factors of delirium after primary total knee replacement surgery | Total knee replacement, USA | CCI  (modified) | ICD | Patients with post-operative delirium had significantly higher CCI scores. Individual comorbidities were entered into multivariate analysis rather than overall CCI score. |  |  |
| Yang,  2023 [137] | 115,147  (0.89%) | Delirium  77 (72–83)  No delirium  70 (62–77)  *median (IQR)* | To explore the incidence of delirium and associated risk factors of delirium after shoulder arthroplasty | ≥18 year olds from National Inpatient Sample database, accounting for approximately 20% of annual hospital admissions,  USA | Number of comorbidities (from pre-defined list of 29) | ICD-9-CM codes | Number of comorbidities was significantly associated with post-operative delirium in univariate analysis and then multivariate analysis with age, sex, race, size and region of hospital, depression, fluid and electrolyte disorders, individual comorbidities, elective admission and private insurance |  |  |
| Zapata,  2022 [138] | 140  (100%) | Psychiatrist in agreement with referrer  81.0 (69.7–89.0)  Not in agreement 73.0 (49.0–83.2)  *median (IQR)* | To determine the discriminating variables associated with delirium diagnosis by a psychiatric compared to the referral diagnosis/reasons given by the referring physicians | Inpatients from a tertiary care teaching hospital, Colombia | CCI-short form | DSM 5 |  | CCI-SF scores were not different between concordant (psychiatrist agreed with referrer) and discordant (psychiatrist diagnosed delirium but this was not mentioned in referral) groups |  |
| Zhang,  2019 [139] | 825  (14.3%) | 79 (65–93)  *median (IQR)* | To determine the incidence and risk factors of post-operative delirium | Hip fracture patients, China | Number of comorbidities (≥2) | DSM 5 | Participants with delirium were significantly more likely to have ≥2 comorbidities. This did not remain significant in multivariate analysis with cognitive impairment, ASA class, transfusion >2 units of red blood cells and ICU admission. |  |  |
| Zhao,  2023 [140] | 199  (28.1%) | 92 (90-94)  *median (IQR)* | To find the prevalence and which variables can predict post-operative delirium in nonagenarians with hip fracture | ≥90 years old with hip fracture,  China | Number of comorbidities (≥4) | CAM | Number of comorbidities ≥4 significantly more likely in delirium versus no delirium groups. Remained significant in multivariate analysis with age, stroke, haemoglobin, albumin, admission to operating time, general versus regional anaesthesia, blood transfusion. |  |  |

Table 1: Summary of all included studies, ordered alphabetically.

Abbreviations: APACHE score=Acute Physiology and Chronic Health Evaluation; ASA=American Society of Anesthesiologists physical status classification system; CAM=Confusion Assessment Method; CAM-ICU=Confusion Assessment Method for the Intensive Care Unit; CCI=Charlson Comorbidity Index; CIRS=Cumulative Illness Rating Scale; CIRS-G=Cumulative Illness Rating Scale – Geriatrics; DOS – Delirium Observation Screening Scale; DRS-R98=Delirium Rating Scale-Revised 98, DSM=Diagnostic and Statistical Manual; HCT-CI=Hematopoietic Cell Transplantation-specific Comorbidity Index; ICD=International Code of Diseases; ICU=Intensive Care Unit; IQR=interquartile range; MDAS=Memorial Delirium Assessment Scale; MMSE=Mini Mental State Examination; SD=standard deviation; USA=United States of America; 4AT=4 A’s Test.

# Table of systematic reviews meeting inclusion criteria

| **Author, year** | **Sample size**  **(% with delirium)** | **Age**  **(mean (SD) unless stated otherwise)** | **Primary objective of the study** | **Setting** | **Measure of MLTC** | **Measure of delirium** | **Impact of MLTC on delirium prevalence** | **Impact of MLTC on delirium presentation** | **The contribution of both delirium and MLTC to outcomes** |
| --- | --- | --- | --- | --- | --- | --- | --- | --- | --- |
| Dasgupta,  2006 [141] | 25 studies included (5.1% to 52.2%) | Not stated | To systematically review commonly assessed pre-operative risk factors for incident delirium | Non-cardiac surgery, Worldwide | Number of comorbidities | DSM or CAM | 8 studies examined the relationship between medical comorbidity and delirium. Comorbidity was measured variably using the Charlson Index, counts of medical conditions and a modified RAND index. Too much missing data in all but 2 studies to pool results. In these two, an association was suggested between increasing numbers of medical conditions and delirium [113]. |  |  |
| Dasgupta,  2010 [142] | 21 studies | All but one study included patients ≥60 years | To review systematically what is known about the factors associated with persistence of delirium | Hospital and rehabilitation patients,  Worldwide | Number of comorbidities and CCI | DSM or a scale derived from DSM |  | CCI found not to be associated with persistent delirium [49] but number of comorbidities was [111]. |  |
| Newman, 2015 [143] | 10 studies (including 100-3570 participants) | 7 studies included older people (>65 or >70 years), 2 included all >18 years and 1 including >50 year olds | To review studies of validated risk-stratification models for delirium | Inclusion criteria: acute medical inpatient population | CCI | DSM or CAM | 1 of the 10 studies included in this review assessed MLTC (measured using CCI) as a risk factor for delirium [49]. |  |  |
| Oh,  2015 [144] | 10 studies (including 34-541 participants) | Review included adults ≥18 years | To systematically identify pre-operative clinical risk factors for incident post-operative delirium in individuals undergoing hip fracture repair | Adult patients (≥18 years) who underwent hip fracture surgery | “multiple comorbidities” – unclear how this was defined | DSM or CAM | Three out of five studies that examined multiple comorbidities found them to be significantly associated with post-operative delirium in the bivariate models [41, 53, 68]. In two of the three, this association was significant in multivariate models. |  |  |
| Raats, 2016 [145] | 10 studies (including 47-463 participants) | Not presented | To systematically review current available literature on pre-operative risk factors for delirium after vascular surgery | Elective vascular surgery patients | CCI | DSM or ICD | Comorbidity was measured using the Charlson Comorbidity Index in three studies [61, 101, 125]. CCI was not confirmed as an independent predictor for delirium. |  |  |
| Rong,  2021 [146] | 11,934 (17.6%) | Not presented | To pool the prevalence and risk factors of post-operative delirium | Hip and knee replacement patients,  Worldwide | CCI | Any validated tool (DSM, CAM, DRS) | CCI, reported by 5 studies [15, 17, 44, 47] was overall higher in the post-operative delirium group compared to the no delirium group. |  |  |
| Sanyaolu,  2020 [147] | 1937 (17%) across 7 articles | 1 x 60 years or older  4 x 65 years or older  1 x 66 years or older  1 x no age restriction | Systematic review and meta-analysis to identify risk factors for delirium in patients undergoing urological surgery | Patients under-going elective or emergency urological surgery, 3xEurope, 2xChina 1xJapan, 1xUSA | Mean age adjusted CCI, CCI>3 or Number of comorbidities ≥2 | Validated delirium diagnostic/  assessment tool (DSM, CAM, ICD) | Comorbidity examined in 4 studies using different methods [45, 67, 122, 134]. Pooling of results suggests a possible association between having ≥2 comorbidities and an increased risk of post-operative delirium. |  |  |
| Yang,  2017 [148] | 5364 (24.0%) | Not presented | To conclude the risk factors of delirium after hip surgery | Hip fracture repair | CCI and “multiple comorbidities” | DSM or CAM | 8 studies used “medical comorbidities” and found it to significantly predict post-operative delirium [68, 80, 113] but with a significant heterogeneity. |  |  |
| Yang,  2021 [149] | 10,053 (8.2%) | Restricted to studies of ≥65 year olds | To quantitively conclude the risk factors for post-operative delirium | Orthopaedic surgery patients,  Worldwide | CCI and disease counts | DSM or CAM | 7 studies included in analysis – pooled analysis found a 2-fold increased risk of delirium in patients with comorbidities [16]. |  |  |

Table 2: Summary of systematic reviews meeting inclusion criteria. Full texts mentioned within these reviews are referenced in the table when they met the inclusion criteria for this scoping review.

Abbreviations: CAM=Confusion Assessment Method; CCI=Charlson Comorbidity Index; DRS=Delirium Rating Scale-Revised 98, DSM=Diagnostic and Statistical Manual; ICD=International Code of Diseases.

# 7. References

1. Abdullah A, Eigbire G, Salama A, et al. Impact of delirium on patients hospitalized for myocardial infarction: A propensity score analysis of the National Inpatient Sample. Clin Cardiol. 2018;41(7):910-5. doi: https://doi.org/10.1002/clc.22972.

2. Afonso A, Scurlock C, Reich D, et al. Predictive Model for Post-operative delirium in Cardiac Surgical Patients. Semin Cardiothorac Vasc Anesth. 2010;14(3):212-7. doi: 10.1177/1089253210374650

3. Alamri SH, Ashanqity OA, Alshomrani AB, et al. Delirium and correlates of delirium among newly admitted elderly patients: a cross-sectional study in a Saudi general hospital. Ann Saudi Med. 2018;38(1):15-21. doi: 10.5144/0256-4947.2018.15.

4. Alvarez EA, Rojas VA, Caipo LI, et al. Non-pharmacological prevention of post-operative delirium by occupational therapy teams: A randomized clinical trial. Front Med (Lausanne). 2023;10:1099594. doi: 10.3389/fmed.2023.1099594.

5. Ansaloni L, Catena F, Chattat R, et al. Risk factors and incidence of post-operative delirium in elderly patients after elective and emergency surgery. Br J Surg. 2010;97(2):273-80. doi: 10.1002/bjs.6843.

6. Arias F, Chen F, Shiff H, et al. Parental Education and Delirium Risk after Surgery in Older Adults. Clin Gerontol. 2023;46(2):253-66. doi: 10.1080/07317115.2022.2111289.

7. Arinzon Z, Peisakh A, Schrire S, Berner YN. Delirium in long-term care setting: indicator to severe morbidity. Arch Gerontol Geriatr. 2011;52(3):270-5. doi: 10.1016/j.archger.2010.04.012.

8. Aziz KT, Best MJ, Naseer ZA, et al. The Association of Delirium with Perioperative Complications in Primary Elective Total Hip Arthroplasty. Clin Orthop Surg. 2018;10:286-91.

9. Bandini M, Marchioni M, Preisser F, et al. Comprehensive analysis of in-hospital delirium after major surgical oncology procedures: A population-based study. Can Urol Assoc J. 2020;14(3):E84-E93. doi: 10.5489/cuaj.6030.

10. Bauernfreund Y, Launders N, Favarato G, Hayes JF, Osborn D, Sampson EL. Incidence and associations of hospital delirium diagnoses in 85,979 people with severe mental illness: A data linkage study. Acta Psychiatr Scand. 2023;147(5):516-26. doi: 10.1111/acps.13480.

11. Béland E, Nadeau A, Carmichael PH, et al. Predictors of delirium in older patients at the emergency department: a prospective multicentre derivation study. Can J Emerg Med. 2021;23(3):330-6. doi: 10.1007/s43678-020-00004-8.

12. Bellelli G, Morandi A, Di Santo SG, et al. "Delirium Day": a nationwide point prevalence study of delirium in older hospitalized patients using an easy standardized diagnostic tool. BMC Med. 2016;14:106. doi: 10.1186/s12916-016-0649-8.

13. Billig AE, Lampert MA, Guerra RR, Steigleder NE. Delirium in the elderly admitted to an emergency hospital service. Rev Bras Enferm. 2022;75Suppl 4(Suppl 4):e20210054. doi: 10.1590/0034-7167-2021-0054.

14. Carrasco MP, Villarroel L, Andrade M, Calderón J, González M. Development and validation of a delirium predictive score in older people. Age Ageing. 2014;43(3):346-51. doi: 10.1093/ageing/aft141.

15. Cerejeira J, Batista P, Nogueira V, Vaz-Serra A, Mukaetova-Ladinska EB. The stress response to surgery and post-operative delirium: evidence of hypothalamic-pituitary-adrenal axis hyperresponsiveness and decreased suppression of the GH/IGF-1 Axis. J Geriatr Psychiatry Neurol. 2013;26(3):185-94. doi: 10.1177/0891988713495449.

16. Chu CS, Liang CK, Chou MY, et al. Short-Form Mini Nutritional Assessment as a useful method of predicting the development of post-operative delirium in elderly patients undergoing orthopedic surgery. Gen Hosp Psychiatry. 2016;38:15-20. doi: 10.1016/j.genhosppsych.2015.08.006.

17. Cunningham EL, McGuinness B, McAuley DF, et al. CSF Beta-amyloid 1-42 Concentration Predicts Delirium Following Elective Arthroplasty Surgery in an Observational Cohort Study. Ann Surg. 2019;269(6):1200-5. doi: 10.1097/sla.0000000000002684.

18. Curyto KJ, Johnson J, TenHave T, Mossey J, Knott K, Katz IR. Survival of hospitalized elderly patients with delirium: a prospective study. Am J Geriatr Psychiatry. 2001;9(2):141-7.

19. Czyzycki M, Klimiec-Moskal E, Chrobak AA, Pera J, Slowik A, Dziedzic T. Subtypes of delirium after ischaemic stroke-predisposing factors and outcomes: a prospective observational study (PROPOLIS). Eur J Neurol. 2022;29(2):478-85. doi: 10.1111/ene.15144.

20. Dasgupta M, Brymer C. Poor functional recovery after delirium is associated with other geriatric syndromes and additional illnesses. Int Psychogeriatr. 2015;27(5):793-802. doi: 10.1017/s1041610214002658.

21. de Haan E, van Rijckevorsel V, Bod P, Roukema GR, de Jong L. Delirium After Surgery for Proximal Femoral Fractures in the Frail Elderly Patient: Risk Factors and Clinical Outcomes. Clin Interv Aging. 2023;18:193-203. doi: 10.2147/cia.s390906.

22. Demirtakan T, Cakmak F, Ipekci A, et al. Clinical assessment and short-term mortality prediction of older adults with altered mental status using RASS and 4AT tools. The Am J Emerg Med. 2024;75:14-21. doi: 10.1016/j.ajem.2023.10.022.

23. Devore EE, Fong TG, Marcantonio ER, et al. Prediction of Long-term Cognitive Decline Following Post-operative delirium in Older Adults. J Gerontol A Biol Sci Med Sci. 2017;72(12):1697-702. doi: 10.1093/gerona/glx030.

24. Di Giorgio A, Mirijello A, De Gennaro C, et al. Factors Associated with Delirium in COVID-19 Patients and Their Outcome: A Single-Center Cohort Study. Diagnostics (Basel). 2022;12(2). doi: 10.3390/diagnostics12020544.

25. Dogrul RT, Dogrul AB, Konan A, et al. Does Preoperative Comprehensive Geriatric Assessment and Frailty Predict Post-operative Complications? World J Surg. 2020;44(11):3729-36. doi: 10.1007/s00268-020-05715-8.

26. Dworkin A, Lee DS, An AR, Goodlin SJ. A Simple Tool to Predict Development of Delirium After Elective Surgery. J Am Geriatr Soc. 2016;64(11):e149-e53. doi: 10.1111/jgs.14428.

27. Eide LS, Ranhoff AH, Fridlund B, et al. Comparison of frequency, risk factors, and time course of post-operative delirium in octogenarians after transcatheter aortic valve implantation versus surgical aortic valve replacement. Am J Cardiol. 2015;115(6):802-9. doi: 10.1016/j.amjcard.2014.12.043.

28. Elder NM, Mumma BE, Maeda MY, Tancredi DJ, Tyler KR. Emergency Department Length of Stay Is Associated with Delirium in Older Adults. West J Emerg Med. 2023;24(3):532-7. doi: 10.5811/westjem.59383.

29. Eschweiler GW, Czornik M, Herrmann ML, et al. Presurgical Screening Improves Risk Prediction for Delirium in Elective Surgery of Older Patients: The PAWEL RISK Study. Front Aging Neurosci. 2021;13:679933. doi: 10.3389/fnagi.2021.679933.

30. Esmaeeli S, Franco-Garcia E, Akeju O, et al. Association of preoperative frailty with post-operative delirium in elderly orthopedic trauma patients. Aging Clin Exp Res. 2022;34(3):625-31. doi: 10.1007/s40520-021-01961-5.

31. Ferré F, Piel-Julian ML, Tincres F, et al. A High Post-operative Atropinic Burden is Associated with Post-operative delirium in Elderly Patients with Hip Fracture: Results of the Prospective, Observational, ATROPAGE Trial. Clin Interv Aging. 2022;17:1931-8. doi: 10.2147/cia.s372400.

32. Fick D, Foreman M. Consequences of not recognizing delirium superimposed on dementia in hospitalized elderly individuals. J Gerontol Nurs. 2000;26(1):30-40. doi: 10.3928/0098-9134-20000101-09.

33. Fineberg SJ, Nandyala SV, Marquez-Lara A, Oglesby M, Patel AA, Singh K. Incidence and risk factors for post-operative delirium after lumbar spine surgery. Spine. 2013;38(20):1790-6. doi: 10.1097/BRS.0b013e3182a0d507.

34. Flaherty JH, Steele DK, Chibnall JT, Vasudevan VN, Bassil N, Vegi S. An ACE unit with a delirium room may improve function and equalize length of stay among older delirious medical inpatients. J Gerontol A Biol Sci Med Sci. 2010;65(12):1387-92. doi: 10.1093/gerona/glq136.

35. Fortes-Filho SQ, Apolinario D, Melo JA, Suzuki I, Sitta Mdo C, Garcez Leme LE. Predicting delirium after hip fracture with a 2-min cognitive screen: prospective cohort study. Age Ageing. 2016;45(5):713-7. doi: 10.1093/ageing/afw084.

36. Franco JG, Trzepacz PT, Sepúlveda E, et al. Delirium diagnostic tool-provisional (DDT-Pro) scores in delirium, subsyndromal delirium and no delirium. Gen Hosp Psychiatry. 2020;67:107-14. doi: 10.1016/j.genhosppsych.2020.10.003.

37. Franz ND, Alaniz C, Miller JT, Farina N. Association Between Sedative Medication Administration and Delirium Development in a Medical Intensive Care Unit. J Pharm Pract. 2023;36(5):1164-9. doi: 10.1177/08971900221096978.

38. Freter S, Dunbar M, Koller K, MacKnight C, Rockwood K. Prevalence and Characteristics of Pre-Operative Delirium in Hip Fracture Patients. Gerontology. 2016;62(4):396-400. doi: 10.1159/000442385.

39. Galanakis P, Bickel H, Gradinger R, Von Gumppenberg S, Förstl H. Acute confusional state in the elderly following hip surgery: incidence, risk factors and complications. Int J Geriatr Psychiatry. 2001;16(4):349-55. doi: 10.1002/gps.327.

40. Giuseffi JL, Borges NE, Boehm LM, et al. Delirium After Transcatheter Aortic Valve Replacement. Am J Crit Care. 2017;26(4):e58-e64. doi: 10.4037/ajcc2017474.

41. Goldenberg G, Kiselev P, Bharathan T, et al. Predicting post-operative delirium in elderly patients undergoing surgery for hip fracture. Psychogeriatrics. 2006;6(2):43-8. https://doi.org/10.1111/j.1479-8301.2006.00146.x.

42. Grover S, Ghormode D, Ghosh A, et al. Risk factors for delirium and inpatient mortality with delirium. J Postgrad Med. 2013;59(4):263-70. doi: 10.4103/0022-3859.123147.

43. Guenther U, Theuerkauf N, Frommann I, et al. Predisposing and precipitating factors of delirium after cardiac surgery: a prospective observational cohort study. Ann Surg. 2013;257(6):1160-7. doi: 10.1097/SLA.0b013e318281b01c..

44. Guo Y, Li Y, Zhang Y, et al. Post-operative delirium associated with metabolic alterations following hemi-arthroplasty in older patients. Age Ageing. 2019;49(1):88-95. doi: 10.1093/ageing/afz132.

45. Hamann J, Bickel H, Schwaibold H, Hartung R, Förstl H. Post-operative acute confusional state in typical urologic population: incidence, risk factors, and strategies for prevention. Urology. 2005;65(3):449-53. doi: 10.1016/j.urology.2004.10.004.

46. Hu XY, Liu H, Zhao X, et al. Automated machine learning-based model predicts post-operative delirium using readily extractable perioperative collected electronic data. CNS Neurosci Ther. 2022;28(4):608-18. doi: 10.1111/cns.13758.

47. Huang J, Bin Abd Razak HR, Yeo SJ. Incidence of post-operative delirium in patients undergoing total knee arthroplasty-an Asian perspective. Ann Transl Med. 2017;5(16):321. doi: 10.21037/atm.2017.06.40.

48. Igwe EO, Ding P, Charlton KE, Nealon J, Traynor V. Association between Malnutrition and Delirium in Older Chronic Kidney Disease Patients Admitted to Intensive Care Units: A Data Linkage Study. J Nutr Health Aging. 2023;27(7):571-7. doi: 10.1007/s12603-023-1938-5.

49. Inouye SK, Zhang Y, Jones RN, Kiely DK, Yang F, Marcantonio ER. Risk factors for delirium at discharge: development and validation of a predictive model. Arch Intern Med. 2007;167(13):1406-13. doi: 10.1001/archinte.167.13.1406.

50. Ito Y, Abe Y, Handa K, et al. Post-operative delirium in Patients after Pancreaticoduodenectomy. Dig Surg. 2017;34(1):78-85. doi: 10.1159/000446928.

51. Janssen TL, Steyerberg EW, Faes MC, et al. Risk factors for post-operative delirium after elective major abdominal surgery in elderly patients: A cohort study. Int J Surg. 2019;71:29-35. doi: 10.1016/j.ijsu.2019.09.011.

52. Jones D, Matalanis G, Mårtensson J, et al. Predictors and Outcomes of Cardiac Surgery-Associated Delirium. A Single Centre Retrospective Cohort Study. Heart Lung Circ. 2019;28(3):455-63. doi: 10.1016/j.hlc.2018.01.007.

53. Juliebø V, Bjøro K, Krogseth M, Skovlund E, Ranhoff AH, Wyller TB. Risk factors for preoperative and post-operative delirium in elderly patients with hip fracture. J Am Geriatr Soc. 2009;57(8):1354-61. doi: 10.1111/j.1532-5415.2009.02377.x.

54. Kang J, An JH, Jeon HJ, Park YJ. Association between ankle brachial index and development of post-operative intensive care unit delirium in patients with peripheral arterial disease. Sci Rep. 2021;11(1):12744. doi: 10.1038/s41598-021-91990-x.

55. Kassie GM, Roughead EE, Nguyen TA, Pratt NL, Kalisch Ellett LM. The Risk of Preoperative Central Nervous System-Acting Medications on Delirium Following Hip or Knee Surgery: A Matched Case-Control Study. Drug Saf. 2022;45(1):75-82. doi: 10.1007/s40264-021-01136-1.

56. Katipoglu B, Naharci MI. Could neutrophil-to-lymphocyte ratio predict mortality in community-dwelling older people with delirium superimposed on dementia? Aging Clin Exp Res. 2022;34(8):1819-26. doi: 10.1007/s40520-022-02108-w.

57. Kennedy M, Enander RA, Tadiri SP, Wolfe RE, Shapiro NI, Marcantonio ER. Delirium risk prediction, healthcare use and mortality of elderly adults in the emergency department. J Am Geriatr Soc. 2014;62(3):462-9. doi: 10.1111/jgs.12692.

58. Khan A, Heslin K, Simpson M, Malone ML. Can Variables From the Electronic Health Record Identify Delirium at Bedside? J Patient Cent Res Rev. 2022;9(3):174-80. doi: 10.17294/2330-0698.1890.

59. Kim J, Park S, Kim KN, et al. Resting-state prefrontal EEG biomarker in correlation with post-operative delirium in elderly patients. Front Aging Neurosci. 2023;15:1224264. doi: 10.3389/fnagi.2023.1224264.

60. Kimura A, Shiraishi Y, Sawamura H, Sugawara R, Inoue H, Takeshita K. Predictors of Post-operative delirium in Older Patients Undergoing Elective Spine Surgery. Spine Surg Relat Res. 2023;7(1):13-8. doi: 10.22603/ssrr.2022-0118.

61. Koebrugge B, van Wensen RJ, Bosscha K, Dautzenberg PL, Koning OH. Delirium after emergency/elective open and endovascular aortoiliac surgery at a surgical ward with a high-standard delirium care protocol. Vascular. 2010;18(5):279-87. doi: 10.2310/6670.2010.00052.

62. Korc-Grodzicki B, Sun SW, Zhou Q, et al. Geriatric Assessment as a Predictor of Delirium and Other Outcomes in Elderly Patients With Cancer. Ann Surg. 2015;261(6):1085-90. doi: 10.1097/sla.0000000000000742.

63. Kroon B, Beishuizen SJE, van Rensen IHT, et al. Delirium in older COVID-19 patients: Evaluating risk factors and outcomes. Int J Geriatr Psychiatry. 2022;37(10). doi: 10.1002/gps.5810.

64. Kuswardhani RAT, Sugi YS. Factors Related to the Severity of Delirium in the Elderly Patients With Infection. Gerontol. Geriatr. Med. 2017;3:2333721417739188. doi: 10.1177/2333721417739188.

65. Lahariya S, Grover S, Bagga S, Sharma A. Delirium in patients admitted to a cardiac intensive care unit with cardiac emergencies in a developing country: incidence, prevalence, risk factor and outcome. Gen Hosp Psychiatry. 2014;36(2):156-64. doi: 10.1016/j.genhosppsych.2013.10.010.

66. Lai CC, Liu KH, Tsai CY, et al. Risk factors and effect of post-operative delirium on adverse surgical outcomes in older adults after elective abdominal cancer surgery in Taiwan. Asian J Surg. 2023;46(3):1199-206. doi: 10.1016/j.asjsur.2022.08.079.

67. Large MC, Reichard C, Williams JT, et al. Incidence, risk factors, and complications of post-operative delirium in elderly patients undergoing radical cystectomy. Urology. 2013;81(1):123-8. doi: 10.1016/j.urology.2012.07.086.

68. Lee HB, Mears SC, Rosenberg PB, Leoutsakos JM, Gottschalk A, Sieber FE. Predisposing factors for post-operative delirium after hip fracture repair in individuals with and without dementia. J Am Geriatr Soc. 2011;59(12):2306-13. doi: 10.1111/j.1532-5415.2011.03725.x.

69. Lee JK, Park YS. Delirium after spinal surgery in Korean population. Spine. 2010;35(18):1729-32. doi: 10.1097/BRS.0b013e3181c423fc.

70. Lee DH, Chang CH, Chang CW, Chen YC, Tai TW. Post-operative delirium in Patients Receiving Hip Bipolar Hemiarthroplasty for Displaced Femoral Neck Fractures: The Risk Factors and Further Clinical Outcomes. J Arthroplasty. 2023;38(4):737-42. doi: 10.1016/j.arth.2022.10.022.

71. Leung JM, Sands LP, Newman S, et al. Preoperative Sleep Disruption and Post-operative delirium. J Clin Sleep Med. 2015;11(8):907-13. doi: 10.5664/jcsm.4944.

72. Li HC, Chen YS, Chiu MJ, Fu MC, Huang GH, Chen CC. Delirium, subsyndromal delirium, and cognitive changes in individuals undergoing elective coronary artery bypass graft surgery. J Cardiovasc Nurs. 2015;30(4):340-5. doi: 10.1097/jcn.0000000000000170.

73. Lim Z, Ling N, Ho VWT, et al. Delirium is significantly associated with hospital frailty risk score derived from administrative data. Int J Geriatr Psychiatry. 2023;38(1):e5872. doi: 10.1002/gps.5872.

74. Lima DP, Ochiai ME, Lima AB, Curiati JA, Farfel JM, Filho WJ. Delirium in hospitalized elderly patients and post-discharge mortality. Clinics (Sao Paulo, Brazil). 2010;65(3):251-5. doi: 10.1590/s1807-59322010000300003.

75. Liu K, Song Y, Yuan Y, et al. Type 2 Diabetes Mellitus with Tight Glucose Control and Poor Pre-Injury Stair Climbing Capacity May Predict Post-operative delirium: A Secondary Analysis. Brain Sci. 2022;12(7). doi: 10.3390/brainsci12070951.

76. Liu J, Li J, He J, Zhang H, Liu M, Rong J. The Age-adjusted Charlson Comorbidity Index predicts post-operative delirium in the elderly following thoracic and abdominal surgery: A prospective observational cohort study. Front Aging Neurosci. 2022;14:979119. doi: 10.3389/fnagi.2022.979119.

77. Lochanie PAN, Ranawaka N. Assessment of incidence and risk factors for intensive care acquired delirium in mechanically ventilated patients in surgical intensive care unit – National Hospital of Sri Lanka. Sri Lankan J. Anaesthesiol. 2018;26(2):131-136. doi: 10.4038/slja.v26i2.8339.

78. Ma IC, Chen KC, Chen WT, et al. Increased Readmission Risk and Healthcare Cost for Delirium Patients without Immediate Hospitalization in the Emergency Department. Clin Psychopharmacol Neurosci. 2018;16(4):398-406. doi: 10.9758/cpn.2018.16.4.398.

79. Mangnall LT, Gallagher R, Stein-Parbury J. Post-operative delirium after colorectal surgery in older patients. Am J Crit Care. 2011;20(1):45-55. doi: 10.4037/ajcc2010902.

80. Marcantonio ER, Flacker JM, Michaels M, Resnick NM. Delirium is independently associated with poor functional recovery after hip fracture. J Am Geriatr Soc. 2000;48(6):618-24. doi: 10.1111/j.1532-5415.2000.tb04718.x.

81. McAlpine JN, Hodgson EJ, Abramowitz S, et al. The incidence and risk factors associated with post-operative delirium in geriatric patients undergoing surgery for suspected gynecologic malignancies. Gynecol Oncol. 2008;109(2):296-302. doi: 10.1016/j.ygyno.2008.02.016.

82. McCullagh IJ, Salas B, Teodorczuk A, Callaghan M. Modifiable risk factors for post-operative delirium in older adults undergoing major non-cardiac elective surgery: a multi-centre, trainee delivered observational cohort feasibility study and trainee survey. BMC Geriatr. 2023;23(1):436. doi: 10.1186/s12877-023-04122-7.

83. McCusker J, Cole MG, Dendukuri N, Belzile E. The delirium index, a measure of the severity of delirium: new findings on reliability, validity, and responsiveness. J Am Geriatr Soc. 2004;52(10):1744-9. doi: 10.1111/j.1532-5415.2004.52471.x.

84. Miao S, Shen P, Zhang Q, et al. Neopterin and mini-mental state examination scores, two independent risk factors for post-operative delirium in elderly patients with open abdominal surgery. J Cancer Res Ther. 2018;14(6):1234-8. doi: 10.4103/0973-1482.192764.

85. Mohanty S, Gillio A, Lindroth H, et al. Major Surgery and Long Term Cognitive Outcomes: The Effect of Post-operative delirium on Dementia in the Year Following Discharge. J Surg Res. 2022;270:327-34. doi: 10.1016/j.jss.2021.08.043.

86. Monacelli F, Signori A, Marengoni A, et al. Delirium and Clusters of Older Patients Affected by Multimorbidity in Acute Hospitals. J Am Med Dir Assoc. 2022;23(5):885-8. doi: 10.1016/j.jamda.2021.10.004.

87. Morandi A, Rebora P, Isaia G, et al. Delirium symptoms duration and mortality in SARS-COV2 elderly: results of a multicenter retrospective cohort study. Aging Clin Exp Res. 2021;33(8):2327-33. doi: 10.1007/s40520-021-01899-8.

88. Moreno-Gaviño L, Ruiz-Cantero A, Bernabeu-Wittel M, et al. Impact of Cognitive Impairment in a Multicentric Cohort of Polypathological Patients. Int J Gerontol. 2012;6:84-9. doi: 10.1016/j.ijge.2011.09.026.

89. Mossello E, Baroncini C, Pecorella L, et al. Predictors and prognosis of delirium among older subjects in cardiac intensive care unit: focus on potentially preventable forms. Eur Heart J Acute Cardiovasc Care. 2020;9(7):771-8. doi: 10.1177/2048872619882359.

90. Naksuk N, Thongprayoon C, Park JY, et al. Clinical impact of delirium and antipsychotic therapy: 10-Year experience from a referral coronary care unit. Eur Heart J Acute Cardiovasc Care. 2017;6(6):560-8. doi: 10.1177/2048872615592232.

91. Narayanan GK, Koshy RC, Amma RO, Subramanian D, Rajendran SP. Incidence of Post-operative delirium and its Association with Intraoperative Blood Pressure Fluctuation in Elderly undergoing Oncosurgery: A Prospective Cohort Study. J Clin Diagn Res. 2022;16(7):UC39-UC45. doi: 10.7860/jcdr/2022/57305.16624.

92. Ng BH, Law ZK, Remli R, et al. Incidence and risk factors of delirium in patients with acute ischaemic stroke. Neurol Asia 2019;24:295–302.

93. O'Keeffe S, Lavan J. The prognostic significance of delirium in older hospital patients. J Am Geriatr Soc. 1997;45(2):174-8. doi: 10.1111/j.1532-5415.1997.tb04503.x.

94. O'Regan NA, Fitzgerald J, Adamis D, Molloy DW, Meagher D, Timmons S. Predictors of Delirium Development in Older Medical Inpatients: Readily Identifiable Factors at Admission. J Alzheimers Dis. 2018;64(3):775-85. doi: 10.3233/jad-180178.

95. Pagali SR, Kumar R, Fu S, Sohn S, Yousufuddin M. Natural Language Processing CAM Algorithm Improves Delirium Detection Compared With Conventional Methods. Am J Med Qual. 2023;38(1):17-22. doi: 10.1097/jmq.0000000000000090.

96. Pasinska P, Kowalska K, Klimiec E, Szyper-Maciejowska A, Wilk A, Klimkowicz-Mrowiec A. Frequency and predictors of post-stroke delirium in PRospective Observational POLIsh Study (PROPOLIS). J Neurol. 2018;265(4):863-70. doi: 10.1007/s00415-018-8782-2.

97. Patil S, Gonuguntala K, Rojulpote C, Kumar M, Corradi JP, Chen K. Delirium is an important predictor of mortality in elderly patients with ST-elevation myocardial infarction: insight from National Inpatient Sample database. Coron Artery Dis. 2020;31(8):665-70. doi: 10.1097/mca.0000000000000978.

98. Pendlebury ST, Lovett NG, Smith SC, et al. Observational, longitudinal study of delirium in consecutive unselected acute medical admissions: age-specific rates and associated factors, mortality and re-admission. BMJ Open. 2015;5(11):e007808. doi: 10.1136/bmjopen-2015-007808.

99. Pioli G, Bendini C, Giusti A, et al. Surgical delay is a risk factor of delirium in hip fracture patients with mild-moderate cognitive impairment. Aging Clin Exp Res. 2019;31(1):41-7. doi: 10.1007/s40520-018-0985-y.

100. Pol RA, van Leeuwen BL, Visser L, et al. Standardised frailty indicator as predictor for post-operative delirium after vascular surgery: a prospective cohort study. Eur J Vasc Endovasc Surg. 2011;42(6):824-30. doi: 10.1016/j.ejvs.2011.07.006.

101. Pol RA, van Leeuwen BL, Izaks GJ, et al. C-reactive protein predicts post-operative delirium following vascular surgery. Ann Vasc Surg. 2014;28(8):1923-30. doi: 10.1016/j.avsg.2014.07.004.

102. Quraishi SA, Litonjua AA, Elias KM, et al. Association between pre-hospital vitamin D status and hospital-acquired new-onset delirium. Br J Nutr. 2015;113(11):1753-60. doi: 10.1017/s0007114515001245.

103. Radinovic K, Markovic-Denic L, Dubljanin-Raspopovic E, Marinkovic J, Milan Z, Bumbasirevic V. Estimating the effect of incident delirium on short-term outcomes in aged hip fracture patients through propensity score analysis. Geriatr Gerontol Int. 2015;15(7):848-55. doi: 10.1111/ggi.12358.

104. Radinovic K, Markovic-Denic L, Milan Z, Cirkovic A, Baralic M, Bumbasirevic V. Impact of intraoperative blood pressure, blood pressure fluctuation, and pulse pressure on post-operative delirium in elderly patients with hip fracture: A prospective cohort study. Injury. 2019;50(9):1558-64. doi: 10.1016/j.injury.2019.06.026.

105. Ranhoff AH, Rozzini R, Sabatini T, Cassinadri A, Boffelli S, Trabucchi M. Delirium in a sub-intensive care unit for the elderly: occurrence and risk factors. Aging Clin Exp Res. 2006;18(5):440-5. doi: 10.1007/bf03324841.

106. Richardson SJ, Davis DHJ, Stephan BCM, et al. Recurrent delirium over 12 months predicts dementia: results of the Delirium and Cognitive Impact in Dementia (DECIDE) study. Age Ageing. 2021;50(3):914-20. doi: 10.1093/ageing/afaa244.

107. Ritchie CW, Newman TH, Leurent B, Sampson EL. The association between C-reactive protein and delirium in 710 acute elderly hospital admissions. Int Psychogeriatr. 2014;26(5):717-24. doi: 10.1017/s1041610213002433.

108. Ritchie C, Walters RW, Ramaswamy S, Alla VM. Impact of delirium on mortality in patients hospitalized for heart failure. Int J Psychiatry Med. 2022;57(3):212-25. doi: 10.1177/00912174211028019.

109. Robinson TN, Raeburn CD, Tran ZV, Angles EM, Brenner LA, Moss M. Post-operative delirium in the elderly: risk factors and outcomes. Ann Surg. 2009;249(1):173-8. doi: 10.1097/SLA.0b013e31818e4776.

110. Romanauski TR, Martin EE, Sprung J, Martin DP, Schroeder DR, Weingarten TN. Delirium in Post-operative Patients Admitted to the Intensive Care Unit. Am Surg. 2018;84(6):875-80. doi: 10.1177/000313481808400635.

111. Rudberg MA, Pompei P, Foreman MD, Ross RE, Cassel CK. The natural history of delirium in older hospitalized patients: a syndrome of heterogeneity. Age Ageing. 1997;26(3):169-74. doi: 10.1093/ageing/26.3.169.

112. Saljuqi AT, Hanna K, Asmar S, et al. Prospective Evaluation of Delirium in Geriatric Patients Undergoing Emergency General Surgery. J Am Coll Surg. 2020;230(5):758-65. doi: 10.1016/j.jamcollsurg.2020.01.029.

113. Schuurmans MJ, Duursma SA, Shortridge-Baggett LM, Clevers GJ, Pel-Littel R. Elderly patients with a hip fracture: the risk for delirium. Appl Nurs Res. 2003;16(2):75-84. doi: 10.1016/s0897-1897(03)00012-0.

114. Sieber FE, Neufeld KJ, Gottschalk A, et al. Effect of Depth of Sedation in Older Patients Undergoing Hip Fracture Repair on Post-operative delirium: The STRIDE Randomized Clinical Trial. JAMA Surg. 2018;153(11):987-95. doi:10.1001/jamasurg.2018.2602.

115. Singler K, Thiem U, Christ M, et al. Aspects and assessment of delirium in old age. First data from a German interdisciplinary emergency department. Z Gerontol Geriatr. 2014;47(8):680-5. doi: 10.1007/s00391-014-0615-z.

116. Smith PJ, Rivelli SK, Waters AM, et al. Delirium affects length of hospital stay after lung transplantation. J Crit Care. 2015;30(1):126-9. doi: 10.1016/j.jcrc.2014.09.010.

117. Son CS, Kang WS, Lee JH, Moon KJ. Machine Learning to Identify Psychomotor Behaviors of Delirium for Patients in Long-Term Care Facility. IEEE J Biomed Health Inform. 2022;26(4):1802-14. doi: 10.1109/jbhi.2021.3116967.

118. Srinonprasert V, Pakdeewongse S, Assanasen J, et al. Risk factors for developing delirium in older patients admitted to general medical wards. J Med Assoc Thai. 2011;94 Suppl 1:S99-104.

119. Sugi T, Enomoto T, Ohara Y, et al. Risk factors for post-operative delirium in elderly patients undergoing gastroenterological surgery: A single-center retrospective study. Ann Gastroenterol Surg. 2023;7(5):832-40. doi: 10.1002/ags3.12676.

120. Tan MC, Felde A, Kuskowski M, et al. Incidence and predictors of post-cardiotomy delirium. Am J Geriatr Psychiatry. 2008;16(7):575-83. doi: 10.1097/JGP.0b013e318172b418.

121. Tkacheva ON, Runikhina NK, Vertkin AL, et al. The diagnosis of delirium in an acute-care hospital in Moscow: what does the Pandora's box contain? Clin Interv Aging. 2017;12:343-9. doi: 10.2147/cia.s123177.

122. Tognoni P, Simonato A, Robutti N, et al. Preoperative risk factors for post-operative delirium after urological surgery in the elderly. Arch Gerontol Geriatr. 2011;52(3):e166-9. doi: 10.1016/j.archger.2010.10.021.

123. van der Sluis FJ, Buisman PL, Meerdink M, et al. Risk factors for post-operative delirium after colorectal operation. Surgery. 2017;161(3):704-11. doi: 10.1016/j.surg.2016.09.010.

124. Villalpando-Berumen JM, Pineda-Colorado AM, Palacios P, Reyes-Guerrero J, Villa AR, Gutiérrez-Robledo LM. Incidence of delirium, risk factors, and long-term survival of elderly patients hospitalized in a medical specialty teaching hospital in Mexico City. Int Psychogeriatr. 2003;15(4):325-36. doi: 10.1017/s104161020300958x

125. Visser L, Prent A, van der Laan MJ, et al. Predicting post-operative delirium after vascular surgical procedures. J Vasc Surg. 2015;62(1):183-9. doi: 10.1016/j.jvs.2015.01.041.

126. Voyer P, McCusker J, Cole MG, St-Jacques S, Khomenko L. Factors associated with delirium severity among older patients. J Clin Nurs. 2007;16(5):819-31. doi: 10.1111/j.1365-2702.2006.01808.x.

127. Voyer P, Cole MG, McCusker J, St-Jacques S, Laplante J. Accuracy of nurse documentation of delirium symptoms in medical charts. Int J Nurs Pract. 2008;14(2):165-77. doi: 10.1111/j.1440-172X.2008.00681.x.

128. Wang L, Seok S, Kim S, Kim K, Lee S, Lee K. The Risk Factors of Post-operative delirium after Total Knee Arthroplasty. J Knee Surg. 2017;30(6):600-5. doi: 10.1055/s-0036-1593872.

129. Wang Y, Yu H, Qiao H, Li C, Chen K, Shen X. Risk Factors and Incidence of Post-operative delirium in Patients Undergoing Laryngectomy. Otolaryngol Head Neck Surg. 2019;161(5):807-13. doi: 10.1177/0194599819864304.

130. Weckmann MT, Gingrich R, Mills JA, Hook L, Beglinger LJ. Risk factors for delirium in patients undergoing hematopoietic stem cell transplantation. Ann Clin Psychiatry. 2012;24(3):204-14.

131. Wetterling T, Junghanns K. Contribution of Different Brain Disorders and Multimorbidity to Delirium Superimposed Dementia (DSD). Geriatrics (Basel). 2023;8(3). doi: 10.3390/geriatrics8030064.

132. Wintermann GB, Weidner K, Strauss B, Rosendahl J. Single assessment of delirium severity during postacute intensive care of chronically critically ill patients and its associated factors: post hoc analysis of a prospective cohort study in Germany. BMJ Open. 2020;10(10):e035733. doi: 10.1136/bmjopen-2019-035733.

133. Witlox J, Kalisvaart KJ, de Jonghe JF, et al. Cerebrospinal fluid β-amyloid and tau are not associated with risk of delirium: a prospective cohort study in older adults with hip fracture. J Am Geriatr Soc. 2011;59(7):1260-7. doi: 10.1111/j.1532-5415.2011.03482.x.

134. Xue P, Wu Z, Wang K, Tu C, Wang X. Incidence and risk factors of post-operative delirium in elderly patients undergoing transurethral resection of prostate: a prospective cohort study. Neuropsychiatr Dis Treat. 2016;12:137-42. doi: 10.2147/ndt.s97249.

135. Yang Q, Wang J, Huang X, Xu Y, Zhang Y. Incidence and risk factors associated with post-operative delirium following primary elective total hip arthroplasty: a retrospective nationwide inpatient sample database study. BMC Psychiatry. 2020;20(1):343. doi: 10.1186/s12888-020-02742-6.

136. Yang Q, Wang J, Chen Y, Lian Q, Shi Z, Zhang Y. Incidence and risk factors of post-operative delirium following total knee arthroplasty: A retrospective Nationwide Inpatient Sample database study. Knee. 2022;35:61-70. doi: 10.1016/j.knee.2022.02.006.

137. Yang Q, Fu J, Pan X, et al. A retrospective analysis of the incidence of post-operative delirium and the importance of database selection for its definition. BMC psychiatry. 2023;23(1):88. doi: 10.1186/s12888-023-04576-4.

138. Zapata C, Garces JJ, Duica K, et al. Variables associated with concordance or discordance for delirium diagnosis between referring and consulting physicians at a Tertiary Hospital in Colombia: Prospective observational study. Medicine (Baltimore). 2022;101(49):e32096. doi: 10.1097/md.0000000000032096.

139. Zhang X, Tong DK, Ji F, et al. Predictive nomogram for post-operative delirium in elderly patients with a hip fracture. Injury. 2019;50(2):392-7. doi: 10.1016/j.injury.2018.10.034.

140. Zhao S, Sun T, Zhang J, Chen X, Wang X. Risk factors and prognosis of post-operative delirium in nonagenarians with hip fracture. Sci Rep. 2023;13(1):2167. doi: 10.1038/s41598-023-27829-4.

141. Dasgupta M, Dumbrell AC. Preoperative risk assessment for delirium after noncardiac surgery: a systematic review. J Am Geriatr Soc. 2006;54(10):1578-89. doi: 10.1111/j.1532-5415.2006.00893.x.

142. Dasgupta M, Hillier LM. Factors associated with prolonged delirium: a systematic review. Int Psychogeriatr. 2010;22(3):373-94. doi: 10.1017/s1041610209991517.

143. Newman MW, O'Dwyer LC, Rosenthal L. Predicting delirium: a review of risk-stratification models. Gen Hosp Psychiatry. 2015;37(5):408-13. doi: 10.1016/j.genhosppsych.2015.05.003.

144. Oh ES, Li M, Fafowora TM, et al. Preoperative risk factors for post-operative delirium following hip fracture repair: a systematic review. Int J Geriatr Psychiatry. 2015;30(9):900-10. doi: 10.1002/gps.4233.

145. Raats JW, Steunenberg SL, de Lange DC, van der Laan L. Risk factors of post-operative delirium after elective vascular surgery in the elderly: A systematic review. Int J Surg. 2016;35:1-6. doi: 10.1016/j.ijsu.2016.09.001.

146. Rong X, Ding ZC, Yu HD, Yao SY, Zhou ZK. Risk factors of post-operative delirium in the knee and hip replacement patients: a systematic review and meta-analysis. J Orthop Surg Res. 2021;16(1):76. doi: 10.1186/s13018-020-02127-1.

147. Sanyaolu L, Scholz AFM, Mayo I, et al. Risk factors for incident delirium among urological patients: a systematic review and meta-analysis with GRADE summary of findings. BMC Urol. 2020;20(1):169. doi: 10.1186/s12894-020-00743-x.

148. Yang Y, Zhao X, Dong T, Yang Z, Zhang Q, Zhang Y. Risk factors for post-operative delirium following hip fracture repair in elderly patients: a systematic review and meta-analysis. Aging Clin Exp Res. 2017;29(2):115-26. doi: 10.1007/s40520-016-0541-6.

149. Yang Y, Zhao X, Gao L, Wang Y, Wang J. Incidence and associated factors of delirium after orthopaedic surgery in elderly patients: a systematic review and meta-analysis. Aging Clin Exp Res. 2021;33(6):1493-506. doi: 10.1007/s40520-020-01674-1.
